# Genome-wide analysis of the *Populus trichocarpa* laccase gene family and functional identification of *PtrLAC23*

**DOI:** 10.3389/fpls.2022.1063813

**Published:** 2023-01-17

**Authors:** Boyang Liao, Chencan Wang, Xiaoxu Li, Yi Man, Hang Ruan, Yuanyuan Zhao

**Affiliations:** ^1^ College of Biological Science and Technology, Beijing Forestry University, Beijing, China; ^2^ National Engineering Research Center of Tree Breeding and Ecological Restoration, Beijing Forestry University, Beijing, China; ^3^ Institute of Environmental Biology and Life Support Technology, School of Biological Science and Medical Engineering, Beihang University, Beijing, China; ^4^ School of Cyber Science and Technology, Beihang University, Beijing, China

**Keywords:** biofuel, *populus trichocarpa*, lignin, laccase, gene family analysis

## Abstract

**Introduction:**

Biofuel is a kind of sustainable, renewable and environment friendly energy. Lignocellulose from the stems of woody plants is the main raw material for “second generation biofuels”. Lignin content limits fermentation yield and is therefore a major obstacle in biofuel production. Plant laccase plays an important role in the final step of lignin formation, which provides a new strategy for us to obtain ideal biofuels by regulating the expression of laccase genes to directly gain the desired lignin content or change the composition of lignin.

**Methods:**

Multiple sequence alignment and phylogenetic analysis were used to classify *PtrLAC* genes; sequence features of *PtrLACs* were revealed by gene structure and motif composition analysis; gene duplication, interspecific collinearity and Ka/Ks analysis were conducted to identify ancient *PtrLACs*; expression levels of *PtrLAC* genes were measured by RNA-Seq data and qRT-PCR; domain analysis combine with cis-acting elements prediction together showed the potential function of *PtrLACs*. Furthermore, Alphafold2 was used to simulate laccase 3D structures, *proLAC23::LAC23-eGFP* transgenic Populus stem transects were applied to fluorescence observation.

**Results:**

A comprehensive analysis of the *P. trichocarpa* laccase gene (*PtLAC*) family was performed. Some ancient *PtrLAC* genes such as *PtrLAC25*, *PtrLAC19* and *PtrLAC41* were identified. Gene structure and distribution of conserved motifs clearly showed sequence characteristics of each *PtrLAC*. Combining published RNA-Seq data and qRT-PCR analysis, we revealed the expression pattern of *PtrLAC* gene family. Prediction results of cis-acting elements show that *PtrLAC* gene regulation was closely related to light. Through above analyses, we selected 5 laccases and used Alphafold2 to simulate protein 3D structures, results showed that *PtrLAC23* may be closely related to the lignification. Fluorescence observation of *proLAC23::LAC23-eGFP* transgenic *Populus* stem transects and qRT-PCR results confirmed our hypothesis again.

**Discussion:**

In this study, we fully analyzed the Populus trichocarpa laccase gene family and identified key laccase genes related to lignification. These findings not only provide new insights into the characteristics and functions of Populus laccase, but also give a new understanding of the broad prospects of plant laccase in lignocellulosic biofuel production.

## 1 Introduction

Biofuels, as a kind of sustainable renewable energy instead of fossil energy, have been widely concerned in recent years. The “second generation” biofuel is made from inedible lignocellulose, through enzymatic hydrolysis of polysaccharides, release of monoses, fermentation, and finally converted into cellulosic ethanol for application (Chang, 2007). Compared with the “first generation” biofuels which produce bioethanol by fermentation of edible parts such as sugar and starches, there is no competition with food supply, and pay more attention to ecological benefits in environmental protection (Prem et al., 2021). According to United States Department of Energy, second-generation biofuels are expected to reduce greenhouse gas emissions by up to 96% (Clark, 2008). The main component of lignocellulosic biomass is plant cell wall, which is composed of cellulose, hemicelluloses and lignin (Cosgrove, 2015). In the process of biofuel conversion, lignin can easily adsorb cellulolytic enzymes and restrict the release of cellulose, thus directly limiting fermentation yield (Zeng et al., 2014). Therefore, changing the content and composition of lignin in plants is an effective measure to boost biofuel production.

Laccase (E.C.1.10.3.2), also known as polyphenol oxidase or urushiol oxidase, was first discovered in the SAP of *Rhus Venicifera* in 1883 (Zoppellaro et al., 2001). As the largest subfamily of multicopper oxidases (LMCOs), laccase has a wide range of substrates and is widely distributed in bacteria, plants, insects and fungi (Mate et al., 2016). Commonly, the molecular size of laccase concentrated in 60-130 KD, composed of 500-600 Aa (Su et al., 2017). Although the amino acid sequence of laccase from different sources is quite different, their catalytic sites are relatively conserved (Janusz et al., 2020). Crystal structure analysis of laccase protein has been found that laccase has three copper ion binding sites (T1, T2, T3) and four copper ions, which can be divided into a type-1 Cu, a type-2 Cu, and two type-3 Cu atoms (Zoppellaro et al., 2001). It is speculated that the catalytic mechanism of laccase is copper ions at the active site of T1 absorb electrons from the reduced substrate, causing the substrate to be oxidized and form free radicals, leading to various non-enzymatic secondary reactions (Sato et al., 2020). The current research on laccase mainly focused on its physicochemical properties and biological activities. Fungal laccase has been widely used *in vitro* lignin degradation (Reyes et al., 2021), wastewater treatment (Unuofin and Okoh, 2019) and dye decolorization (Ahmed et al., 2020). Laccase CotA in *Bacillus subtilis* has been reported to play a role in pigment synthesis and protection against ultraviolet damage (Hullo et al., 2001). Insect laccase has been shown to be associated with cuticle formation in insects (Asano et al., 2019). However, the function of plant laccase need to be more determined. Several lines of evidence suggest that laccase can play a key role in plant lignification, which refers to the coupling of a lignin monomer with the terminal of a growing polymer (Bao et al., 2016). Lignin monomers, also called monolignols, are the non-methoxylated *p*-coumaryl alcohol, the monomethoxylated coniferyl alcohol and the dimethoxylated sinapyl alcohol, which forms p-hydroxyphenyl (H), guaiacyl (G) and syringyl (S) units in lignin polymer, respectively. Laccase can catalyze the oxidative dehydrogenation of lignin monomers to form free radicals, once the radicals are generated, polymerization of lignin monomers will take place in a pure chemical reaction, no longer catalyzed by enzymes or proteins (Ralph et al., 2019).

There has been several evidence that laccase is closely associated with lignification. Berthet et al. have found that *LAC4* and *LAC17* played important roles in the lignification process of *Arabidopsis* stems, silencing of these two genes significantly reduced the biosynthesis of lignin (Berthet et al., 2011). In addition, simultaneous knockout *LAC11*, *LAC4*, and *LAC17* in *Arabidopsis* severely affected plant growth, resulting in narrowing of root diameter, closure of anthers, stagnation of vascular tissue development, and a significant reduction in lignification (Zhao et al., 2016). Richard Dixon et al. have reported that GhLAC8, a laccase specifically expressed in the seed coats of *Cleome hassleriana*, which can facilitate the polymerization of lignin in plants by using caffeyl alcohol as the sole substrate, and determined the lignin component content in plant cell walls (Wang et al., 2020). Except for the role in lignification, laccase has many other functions also worth exploring (Wang et al., 2015a). Results show that expression of *GhLac1* in transgenic cotton could enhance the defense response of cotton to against pathogens and pests (Acid et al., 2018). Almost all of the *GmLAC* genes in soybeans are involved in responding to *P.sojae* infection (Wang et al., 2019). Some researchers inserted *Arabidopsis* laccase gene *AtLAC15* promoter into *Brassica napus*, and found that *AtLAC15* promoter could be used as a seed coat-specific promoter for canola. In addition, this coat-specific promoter can help to overexert or inhibit the expression of some genes, which can alter the metabolism and producing seed with reduced fiber content (Wu and Saleh, 2011).

In recent years, Batch anaerobic digestion experiments on 41 energy crops showed that 80% of the sample biofuel yield variation could be explained by lignin content (Dandikas et al., 2014). In addition, inhibition of lignin biosynthesis pathway gene *Pt4CL1* in transgenic poplar (*Populus tremuloides Michx.*) showed that there is a compensatory mechanism between lignin and cellulose, that is, inhibition of lignin synthesis can increase the accumulation of cellulose (Hu et al., 1999). From the perspective of energy utilization, treatment of laccase in plants can inhibit lignin deposition and increase cellulose content, which provides a new idea for the improvement of energy plants (Mai and Kharazipour, 2001).

As an important biomass energy source (Dandikas et al., 2014), woody plant is of great significance to analyze the function and mechanism of its laccase gene. To date, the *Populus trichocarpa* laccase gene family members have been identified (Lu et al., 2013) (Bryan et al., 2016), but their sequence characteristics, structures and functions have not been elucidated in detail. Here, we choose *Populus trichocarpa* as the research object, through collinearity analysis, gene structure analysis, motif and domain analysis, expression pattern analysis, cis-acting elements and protein structure prediction and other detailed analyses of *Populus trichocarpa* laccase gene family, to find out some *PtrLACs* which involved in plant lignification, in order to provide more directions for improvement of energy trees.

## 2 Methods

### 2.1 Multiple sequence alignment, phylogenetic analysis, and classification of laccase family genes

The genome of *Populus trichocarpa v3.0* and *Arabidopsis* were downloaded from Phytozome (https://phytozome.jgi.doe.gov) (Goodstein et al., 2012). According to published research, *Arabidopsis thaliana* has a total of 17 laccase genes (Mccaig et al., 2005), this number is 5 in *Zea mays* (Caparrós-Ruiz et al., 2006), and 53 in *Populus trichocarpa* (Bryan et al., 2016). Each of the 75 sequences has three domains, which named Cu-oxidase, Cu-oxidase_2, and Cu-oxidase_3 (PF00394, PF07731, and PF07732). An alignment of 17 AtLAC peptides, 5 ZmLAC peptides,53 PtrLAC peptides, 46 P*. deltoides* laccase peptides and 12 P*. tomentosa* laccase peptides were performed using ClustalW in MEGA 10.0 with the default parameters. Full-length sequences of all proteins were used to construct phylogenetic tree with the neighbor-joining(NJ) method (1000 replicates) (Kumar et al., 2018). Finally, the tree was annotated and decorated by ITOL online tools (http://itol.embl.de/) (Letunic and Bork, 2016).

### 2.2 Gene structure, motif composition and domain analysis

The evolutionary tree construction method of 53 genes was the same as shown in 2.1. The exon-intron organization of laccase genes was identified by the online program Gene Structure Display Server 2.0 (http://gsds.cbi.pku.edu.cn) (Hu et al., 2015). The MEME online program (http://meme.nbcr.net/meme/intro.html) was used to identify conserved motifs in *Populus trichocarpa* laccases (Bailey et al., 2009), and the maximum number of motif was set to 15. The domain information of each *Populus* laccase protein was obtained from Pfam database (https://pfam.xfam.org/search). Visualization of the motif and domain position was accomplished by TBtools software (https://github.com/CJ-Chen/TBtools) (Chen et al., 2020).

### 2.3 Chromosomal localization and gene duplication

Chromosome length and the distribution of 53 *LAC* genes of *P. trichocarpa* were obtained by using the Phytozome database (https://phytozome.jgi.doe.gov). Multiple Collinearity Scan toolkit (MCScanX) (Wang et al., 2012) was adopted to analyze the gene duplication events with the default parameters. The Gene Location Visualize tool of TBtools software (https://github.com/CJ-Chen/TBtools) was used to Visualize the results of tandem repeat analysis, the Circle Gene View function in TBtools was used to exhibit the collinearity of laccase gene family in *P. trichocarpa*, the *Dual Systeny Plot* in TBtools was used to show the synteny relationships between *P. trichocarpa* and other 5 species. Ka/Ks Calculator 2.0 was used to calculate the Ka/Ks ratio of *PtrLACs* to reveal the natural selection pressure experienced by laccase genes during the evolution (Wang et al., 2010).

### 2.4 Expression pattern of *PtrLACs*

The RNA-Seq data and FPKM (Fragments per kilobase of transcript per million fragments mapped) values of 40 samples including different tissues and developmental stages were obtained from phytozome database. The expression level of *PtrLAC* genes were presented in the form of heatmap by TBtools software.

### 2.5 Analysis of cis-acting elements in *PtrLAC* genes’ promoters

The upstream sequences (2000 bp) of 53 *PtrLAC* genes were extracted by PGSC. Then, PlantCare (http://bioinformatics.psb.ugent.be/webtools/plantcare/html/) was used for predictive analysis (Lescot et al., 2002), and 9 cis-acting elements were obtained after screening. They are abscisic acid responsiveness, auxin responsiveness, defense and stress responsiveness, drought-inducibility, gibberellin-responsiveness, light responsiveness, low-temperature responsiveness, MeJA-responsiveness, MYB binding site involved in light responsiveness.

### 2.6 Properties of *Populus trichocarpa* laccase proteins

The number of amino acids per protein was obtained using the phytozome database, the molecular weight (Da) and isoelectric point (pl) of the protein were predicted by using the online ExPASy tool (https://web.expasy.org/protparam/) (Gasteiger et al., 2003). Subcellular locations of PtrLAC members were calculated by the online software CELLO (http://cello.life.nctu.edu.tw/).The signal peptides of PtrLACs were predicted by using the SignalP online tool (http://www.cbs.dtu.dk/services/SignalP/) (Almagro Armenteros, 2019).

### 2.7 Structural prediction of laccase proteins

Protein structure was predicted by AlphaFold2. The structure prediction process was roughly as described in AlphaFold Paper 2, consisting of five steps:MSA construction, template search, five model reasoning, average PLDD-based model ordering, and constraint relaxation of the predicted structure. Structure factors of ZmLAC3, which coded as 6KLG (ZmLAC3 native), 6KLI (ZmLAC3–SinA complex) and 6KLJ (ZmLAC3–ConA complex), were downloaded from Protein Data Bank (http://www.rcsb.org/) (Andreaeopsida, 2013). AutoDock Vina software were used for molecular docking of PtrLAC with SinA and ConA (Trott and Olson, 2009). Structural comparison between ZmLAC3 and PtrLACs as well as Polar interaction between monolignols and amino acid were analyzed by PyMol 2.0 (Seeliger and De Groot, 2010).

### 2.8 Plant materials

45-day-old 84K poplars (*P.alba×P.glandulosa*) were used as materials for qRT-PCR. Primers gaattcgagctcGGTACCattcaaacctgcgttgatcc and aagcttgcatgcCTGCAGacacttgggaagatcagatggtgg were used to clone a 4.7 kb genomic region into *pCAMBIA1305.1-native-eGFP* for generating *proLAC23::LAC23-eGFP*. 2-mo-old *pLAC23-LAC23-EGFP* line was used for fluorescence observation.

### 2.9 Fluorescence microscope observation

The stem transverse section preparation was followed as described by Li et al. (Li et al., 2011). Images were observed and captured by using the LEICA DM2500 fluorescence microscope. The autofluorescence of lignin was observed using the third gear (UV excitation light) of the microscope, and EGFP fluorescence was observed using the fifth gear (blue excitation light) of the microscope.

### 2.10 Quantitative Real-Time PCR of laccase transcript abundance

Total RNA was isolated from young leaves, young stems and young roots. cDNA of these three samples were obtained by reverse transcription. Gene-specific forward and reverse primers (**Table S1**) were used for quantitative RT-PCR to estimate the abundance of gene-specific laccase transcripts in different tissues and stages. The *SuperReal PreMix Plus (SYBR Green)* kit used was purchased from Tiangen Biotech (Beijing) Co., LTD. The instrument used in the qRT-PCR experiment was Bio-Red.

## 3 Results

### 3.1 Chromosomal location, duplication and expansion events of *Populus* laccase gene family

**Figure 1:**
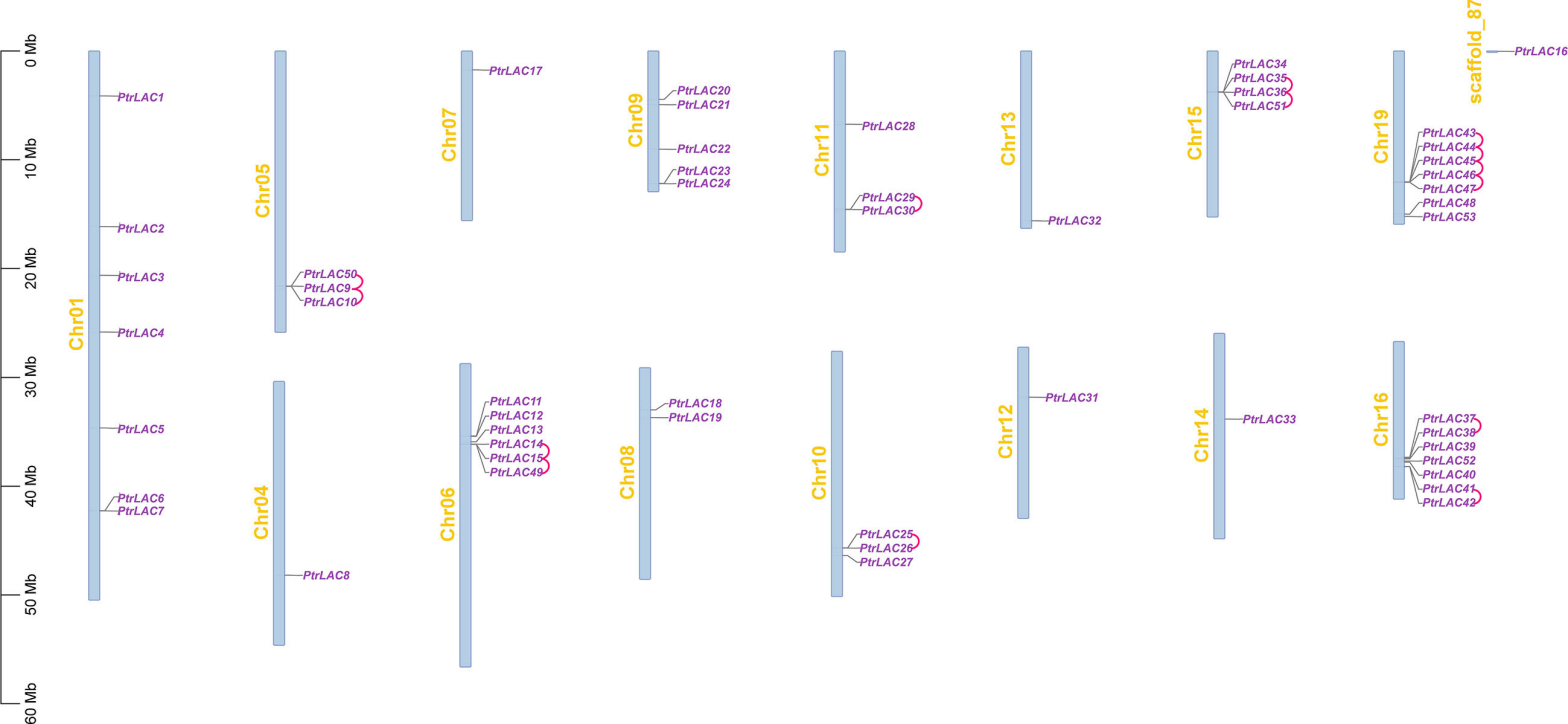
Chromosomal location and gene duplication of *P. trichocarpa* laccase. The tandem duplicated genes are connected by red curves.

It has been reported that there are 53 laccase genes in *Populus trichocarpa*, which are located on 16 chromosomes (**Figure 1**). Most of the laccase genes are concentrated on five chromosomes (chr 01, chr 06, chr 09, chr 16 and chr 19), and some chromosomes have only one gene (chr 04, chr 07, chr 12, chr 13, chr 14 and scaffold_87).

**Figure 2:**
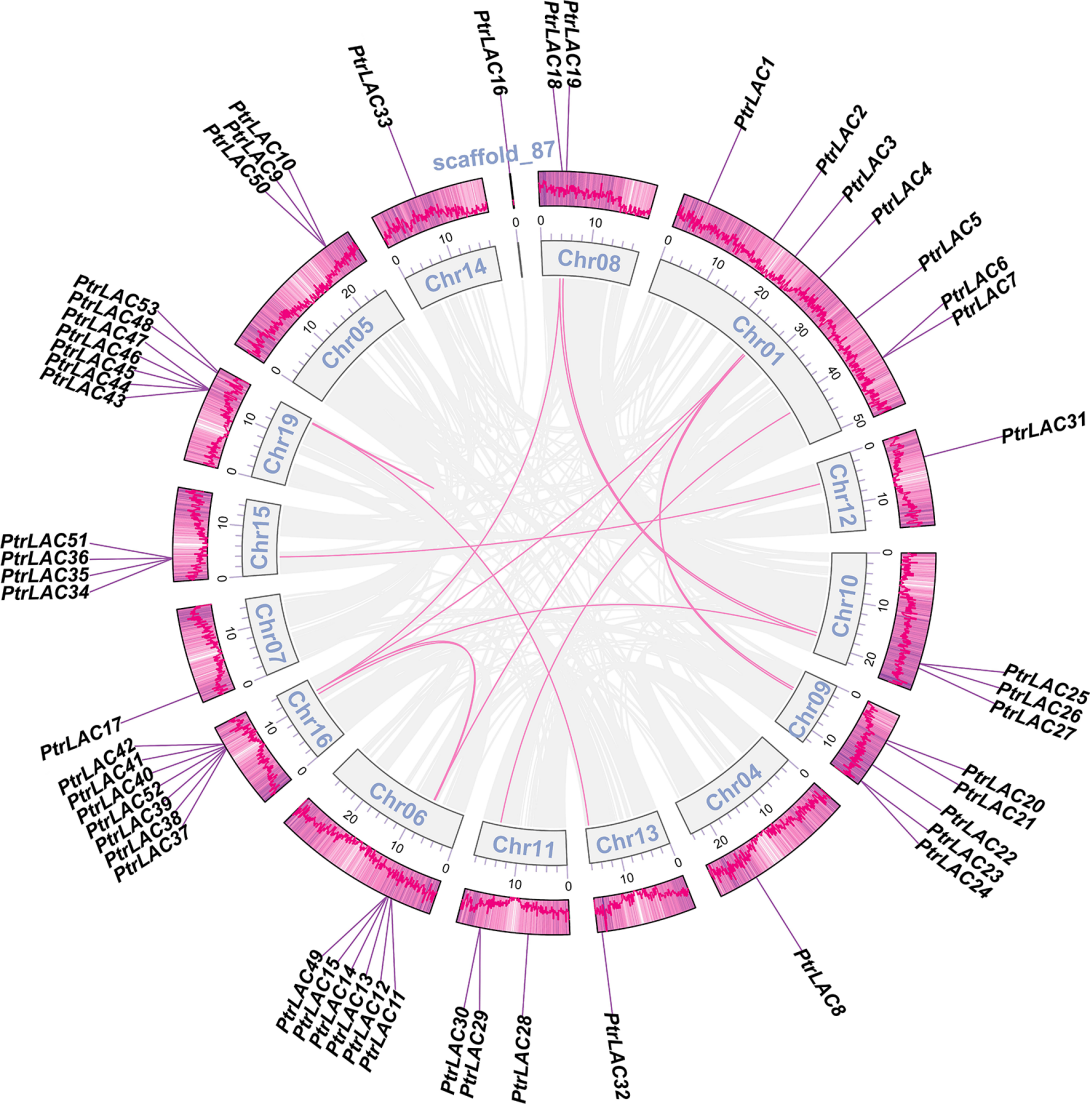
Collinearity of the *PtrLAC* gene pairs. The outermost scale shows gene density on each chromosome, genes are listed in the outer circle according to their chromosomal location. The inner circle represents the chromosomes. Gray lines indicate all synteny blocks in the *Populus trichocarpa* genome, synteny relationships between gene pairs are marked with a pink line.

Segmental and tandem duplications are usually considered to be the main causes of large gene family expansion in plants (Cannon et al., 2004), thus we analyzed the tandem and segmental duplication events of *PtrLACs* family. The results showed that 22 genes (41.5%) were considered as tandem duplicates, among which two pairs of independent tandem duplication genes were located in chr 10, chr 11 and chr 16. Three groups of three tandem duplicated genes located on chr 05, chr 06 and chr 15. Five tandem duplicated genes located on chr 19. Segmental duplications are regions of 1000 or more base pairs with a DNA sequence identity of 90% or greater that are present in more than one copy (Giannuzzi et al., 2011)^-^ (Van de Peer et al., 2009). Therefore, the collinearity gene pair can be considered as the fragment duplication genes in *Populus* laccase gene family. In our study, a total of 22 genes (41.5%) were involved in fragment replication (**Figure 2**). Based on above results, it could be inferred that tandem duplication and fragment replication play an equal important role in the expansion of *Populus* laccase gene family.

To better understand the evolutionary constraints acting on *Populus* laccase gene family, we calculated the non-synonymous nucleotide substitution rate (Ka) and synonymous nucleotide substitution rate (Ks) between both segmental and tandem duplicated *PtrLAC* gene pairs (So, 2002). The Ka/Ks value of almost all of the gene pairs was less than one (except the tandem repeat pair *PtrLAC45*/*PtrLAC46*), which indicated that *Populus* laccase gene family underwent a strong purifying selective pressure during the evolution.

Existing molecular evolution analysis combined with fossil evidence shows that the molecular evolution rate (E value) of *Populus* is about 1/6 of that of *Arabidopsis* (Tuskan and Torr, 2012), while the E value of *Arabidopsis* is 1.5×10^-8^ replacement/synonymous replacement site/year (Koch et al., 1998). Therefore, the E value of *Populus* in this paper is 1/6 of that of *Arabidopsis*, that is, 2.5×10^-9^ replacement/synonymous replacement site/year. Salicoid duplication event occurs between about 60 and 65 MYA (Million Years Ago) (Tuskan and Torr, 2012). Estimation of the repetition time of collinearity gene pairs in *PtrLAC* gene family show that about 13.3% (4/30) gene pairs were separated during the WGD event, 20.0% (6/30) of the repeats were replicated earlier than 150 million years ago, and the rest collinear gene pairs were replicated after the WGD events. In addition, tandem repeats were often replicated later than segmental repeats, suggesting that fragment duplication and tandem duplication were the main driving forces of gene family expansion in the early and late stages, respectively (**Table S2, S3**). These analyses helped us screen out some ancient laccase genes, such as *PtrLAC4, PtrLAC41, PtrLAC19, PtrLAC25* and *PtrLAC18*.

### 3.2 General information, phylogenetic analysis and classification of *PtrLAC* genes

**Figure 3:**
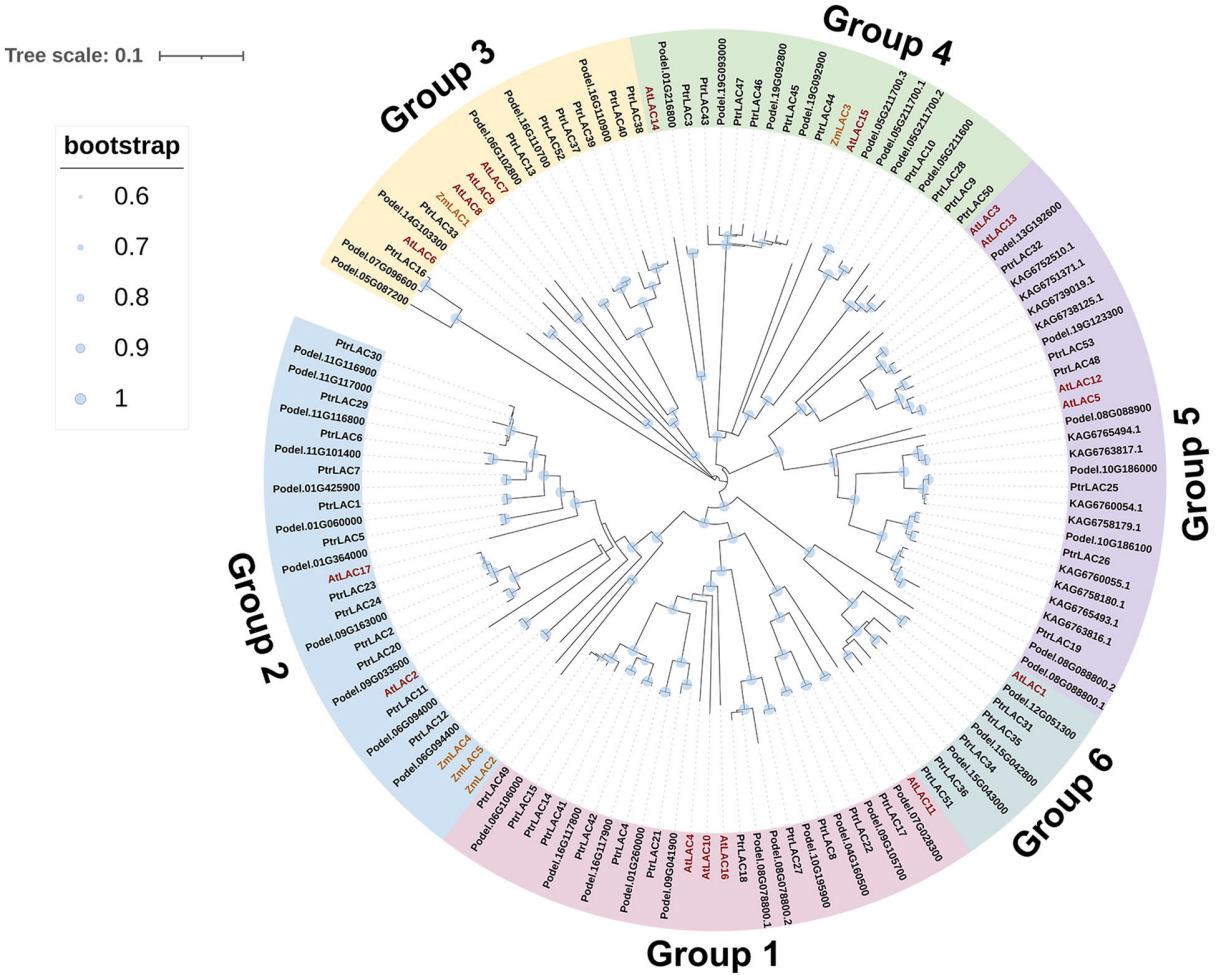
Phylogenetic tree of laccase proteins from *Arabidopsis thaliana* TAIR10 (AtLAC), *Zea mays* (ZmLAC), *Populus trichocarpa* v3.0 (PtrLAC), *Populus deltoides* WV94 v2.1 (Podel) and *Populus tomentosa* (KAG). Full length amino acid sequences of these 133 proteins were aligned using the ClustalW program. Nneighbor-joining tree was constructed using MEGA10.0. 6 groups were distinguished in different colors.

To study the evolutionary relationships among the 75 laccase genes in *Populus Arabidopsis*, and maize, we built a phylogenetic tree using full-length amino acid sequences. In total, 53 sequences from*P. trichocarpa*, 17 sequences from *Arabidopsis*, 5 sequences from maize, 46 sequences from *P. deltoides*, and 12 sequences from *P. tomentosa* were assessed in the phylogenetic tree (**Figure 3**). The phylogenetic analysis indicated that *PtrLACs* could be divided into six large groups corresponding to group 1 to 6 in *Arabidopsis* as defined by Bonnie C (Mccaig et al., 2005). The group 1, 2 and 4 each contains 12 *PtrLAC* members, group 3 and group 5 each possesses 6 members, and group 6 contains 5 members. In addition, we found that members of group 4 involved in tandem duplication accounted for 66.7% (8/12), all the genes belong to group 5 and group 6 were involved in gene duplication events. These phenomena suggest that gene duplication is responsible for expansion of these 3 subfamilies.

The subcellular prediction shows that most of the laccase proteins are likely to be extracellular, but some have been shown to be localized on the cell membrane or in lysosomes and peroxisomes. A signal peptide analysis shows that most laccases are secretory proteins and have a signal peptide of about 27 amino acids, but the signal peptide of PtrLAC51 was not predicted, indicating that it may be intracellularly localized (**Table S4**). We found that most laccases in poplar are alkaline, which is similar to the results in most plants, such as *A. thaliana* and maize.

### 3.3 Exon-intron structure, motif distribution and domain composition of *Populus* laccase gene family

**Figure 4:**
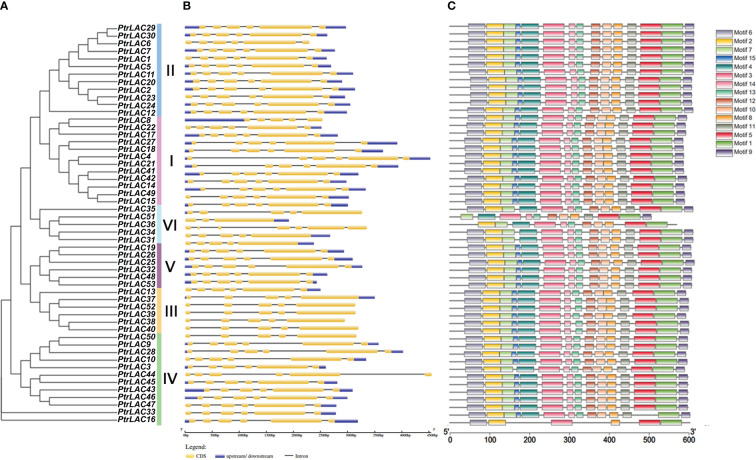
Phylogenetic relationships, gene structure and architecture of conserved protein motifs of *P. trichocarpa* laccase gene family. **(A)** Nneighbor-joining tree was constructed based on the full-length sequences of *P. trichocarpa* laccase proteins using MEGA 10.0 software. **(B)** Exon-intron structure of *P. trichocarpa* laccase genes. Blue boxes indicate untranslated 5′- and 3′-regions; yellow boxes indicate exons while black lines indicate introns. **(C)** The motif composition of *Populus trichocarpa* laccase proteins. Colored boxes represent the different motifs, indicated in the top right-hand corner. The scales at the bottom of the image indicate the estimated exon/intron length in kb and motif length in numbers of amino acids (aa), respectively.

To better understand the structural features of *PtrLACs* and gain more insight into the evolution of the laccase family in *P. trichocarpa*, we analyzed the intron-exons composition of all sequences as shown in **Figure 4**. All the sequences in the group 1, group 3 and group 4 contains 5 introns and 6 exons, most sequences in group 2 also have such structure, except *PtrLAC8*, which contains 4 introns and 5 exons. The sequences in group 5 and most of the sequences in group 6 have four introns and five exons. Notably, in group 6, *PtrLAC51* contained only 3 exons and 2 introns, while *PtrLAC36* contained 7 exons and 6 introns. Overall, the family members with closer genetic relationships have more similar gene structures (**Figure 4B**).

To further clarify the characteristics of the *Populus* laccase family, we detected 15 conserved motifs in 53 sequences and analyzed their distribution in combination with the evolutionary tree. As expected, most of the laccase protein sequences in *Populus trichocarpa* had all 15 motifs. However, in group 6, none of the sequences had motif 15. In addition, *PtrLAC51* also lost motif 2 and motif 6, *PtrLAC36* doesn’t include motif 6, 9 and 11. In group 4, *PtrLAC3* doesn’t have motif 15, and *PtrLAC16* just contains six motifs (motif 6, 2, 3, 8, 5 and 1). These differences suggest that family members of the two branches may have lost part of their motifs during evolutionary process, resulting in a new function. (**Figure 4C**).

**Figure 5:**
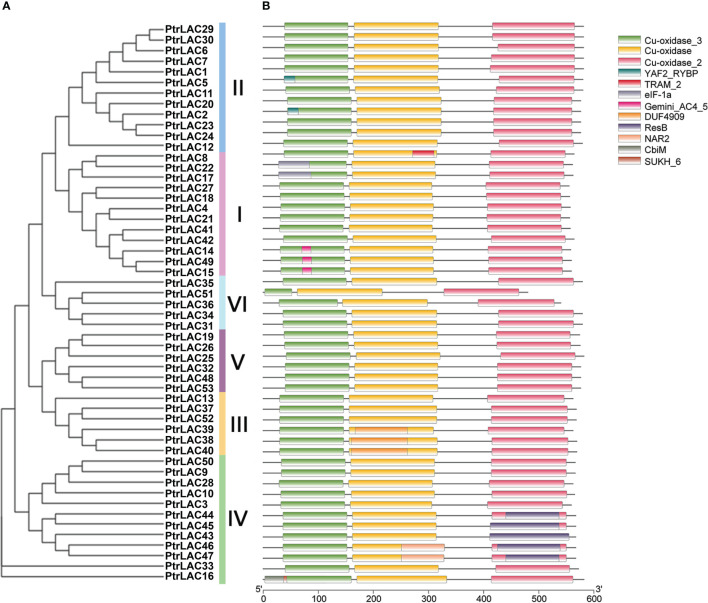
Phylogenetic relationships and conservative domain distribution of the *PtrLACs* family. **(A)** Nneighbor-joining tree was constructed based on the full-length sequences of *P. trichocarpa* laccase proteins using MEGA 10.0 software. **(B)** Domain distribution of *P. trichocarpa* laccase proteins. Heterochromatic boxes represent different domains, indicated in the top right-hand corner. The length of protein can be estimated using the scale at the bottom.

In order to define the function of each member of the *Populus* laccase gene family, we use the Pfam database (https://pfam.xfam.org/search) to analyze the domain of each sequence. Results showed that all the members have Cu-oxidase, Cu-oxidase_2, and Cu-oxidase_3 (PF00394, PF07731, and PF07732) these three conserved domains, which associated with the redox reaction of substrates. Beyond that, we also detected 9 domains and some of them have specific functions: YAF2_RYBP was a C-terminal binding motif, which is first found in YAF2 and RYBP proteins and usually forms the RYBP -/YAF2-PRC1 complex (Wang et al., 2015b); ResB domain was related to the synthesis of cytochrome C and is essential for plant growth (Le Brun et al., 2000); NAR2 is a plant protein with a C-terminal transmembrane region, which often works with NRT2 and can transport nitrate at low concentration, this NAR2-NRT2 system plays an important role in the regulation of lateral root growth (Yong et al., 2010); CBiM is a substrate specific component of the cobalt transport complex CBiMNQO, which is related to the synthesis of coenzyme B12 (Santos et al., 2008) (**Figure 5**). Laccases with these special domains may enable to perform other specific functions in plants, and should be the focus of future research.

### 3.4 Synteny analysis of *PtrLAC* genes

**Figure 6:**
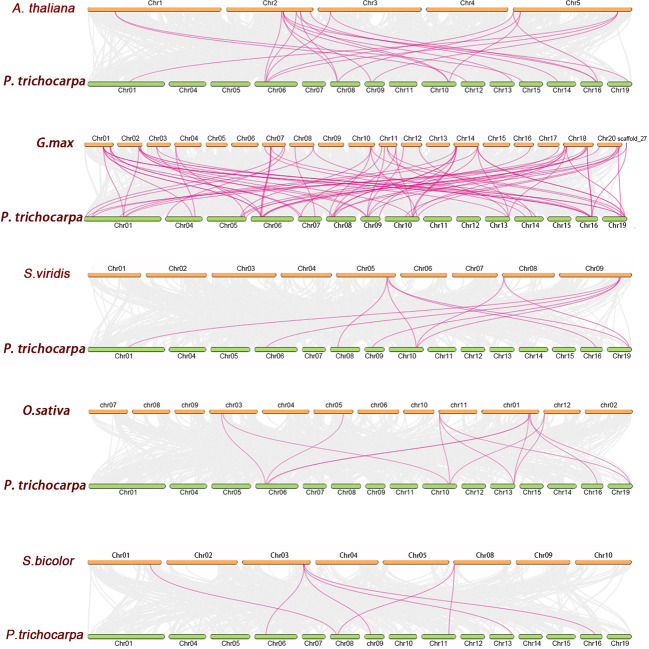
Synteny analysis of *LAC* genes between *P. trichocarpa* and five representative plant species. Gray lines indicate the collinear blocks within *P. trichocarpa* and other plant genomes, while the purple lines highlight the syntenic *LAC* gene pairs.

To further reveal the phylogenetic mechanisms of *Populus* laccase gene family, we constructed five comparative syntenic maps of *Populus* associated with five representative species, including three monocots (*Setaria viridis*, *Oryza sativa*, *Sorghum bicolor*) and two dicots (*Arabidopsis thaliana* and *Glycine max*). Results show that, 16 *PtrLACs* had a collinear relationship with *Arabidopsis thaliana*, and the number was 5, 7, 6 and 24 for sorghum, rice, *Setaria* and soybean. As shown in **Figure 6**, the number of orthologous gene pairs between *Populus* and other species was 22 gene pairs (with *Arabidopsis*), 10 gene pairs (with *Setaria*), 13 gene pairs (with rice), 7 gene pairs (with sorghum) and 59 gene pairs (with soybean), respectively. Some *PtrLAC* genes were found to be associated with at least three syntenic gene pairs, such as *PtrLAC19* (between *Populus* and *Arabidopsis*), *PtrLAC25* (between *Populus* and *Setaria*), *PtrLAC25* and *PtrLAC32* (between *Populus* and Rice). This phenomenon is particularly prominent between *Populus* and soybean, there are 9 collinearity gene pairs of this type in between (*PtrLAC4, PtrLAC50, PtrLAC3, PtrLAC19, PtrLAC21, PtrLAC25, PtrLAC32, PtrLAC37, PtrLAC48*).

We also found that, there were 59 collinear gene pairs of laccase genes between *Populus* and soybean, but only 7 between *Populus* and *Sorghum*, which may relate to the phylogenetic relationships between *Populus* and other five plant species. What is noteworthy is that, many *PtrLACs* (*PtrLAC4, PtrLAC21, PtrLAC33, PtrLAC48*) that are collinearly related to *Arabidopsis* and soybean have not been found in other three monocotyledons, which may indicate that these orthologous pairs formed after the divergence of dicotyledonous and monocotyledonous plants. Some other *PtrLACs*, however, were identified have collinearity with at least four species (*PtrLAC11, PtrLAC25, PtrLAC20, PtrLAC19*), suggesting that these genes maybe present before the ancestors differentiated.

### 3.5 Expression patterns of *PtrLAC* genes

**Figure 7:**
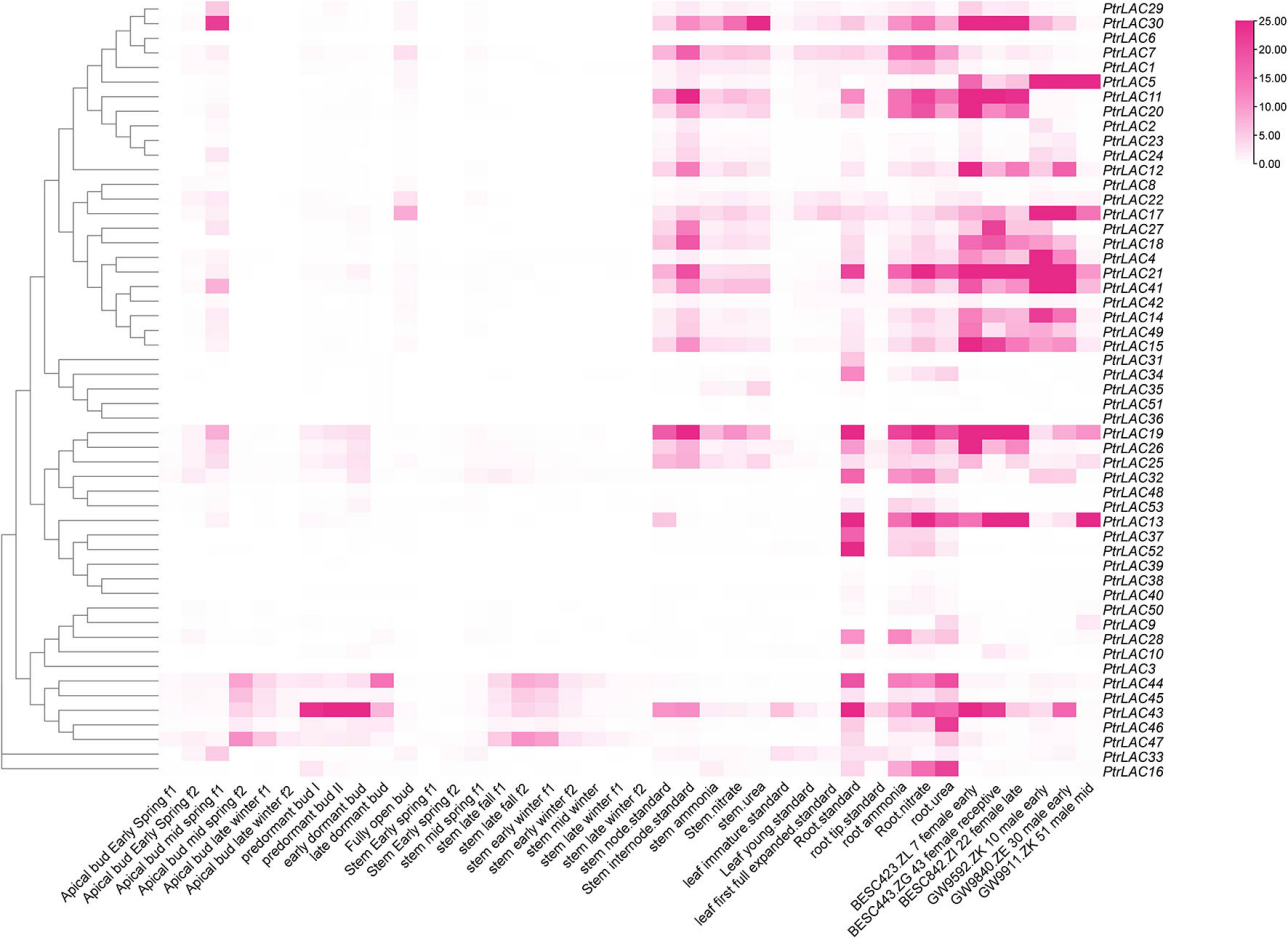
Expression profiles of *PtrLAC* genes in 40 samples including different tissues and developmental stages.

In order to understand the expression of *PtrLACs* in different tissues and stages, we use the RNA-seq data published in Phytozome to draw a heat map and analyzed its FPKM (Fragments Per Kilobase Million) values. we compared the expression patterns of *PtrLACs* in different developmental processes such as bud set and bud flush, male/female catkin development, leaf expansion and root/stem response to different nitrogen nutrition as shown in **Figure 7**.

At different stages of apical bud growth, the expression of laccase genes was more active in mid-spring. During the formation of dormant buds, the expression level of *PtrLAC43* is higher in the pre-dormant buds and early dormant buds, which may be related to the protection of meristem in winter. In general, most *PtrLAC* genes were expressed at low levels in the apical meristem of *Populus*, which was consistent with the results in *Arabidopsis*. High expression level of laccase genes was not detected at different stages of stem development, but it was observed in some members of the group 4 (*PtrLAC44, PtrLAC45, PtrLAC43, PtrLAC47*) that the expression level was higher in late fall f2 and early winter f1, indicating a dynamic response to seasonal variation. It is worth noting that the expression levels of most *PtrLACs* are high in both nodes and internodes, which may be related to the involvement of laccase in the lignification and thickening of the plant secondary wall.

In male catkin development, *PtrLAC5* is consistently highly expressed, most laccase genes had high expression in the early stage of male catkin development and 3 genes (*PtrLAC13, 19, 25*) showed high expression level in the middle development of male catkin, showed dynamic changes of transcription abundance. In female catkin development, compared with other laccase genes, *PtrLAC30, 11, 21, 19* showed consistently higher expression, most of the *PtrLACs* showed certain dynamic changes with the developmental stage.

In addition, we also analyzed the expression of *PtrLAC* genes under different nitrogen sources. The results showed that *PtrLAC* gene was not significantly differentially expressed in stems due to different nitrogen sources, while in roots, it was found that the expression levels of some laccase genes were significantly increased under nitrate nitrogen treatment compared with other nitrogen sources, such as *PtrLAC11, PtrLAC13, PtrLAC14, PtrLAC18, PtrLAC19, PtrLAC20, PtrLAC21, PtrLAC26*, and *PtrLAC41*. Accordingly, we speculated that most *PtrLAC* genes may be involved in the assimilation of nitrate nitrogen in roots (**Figure 7**).

**Figure 8:**
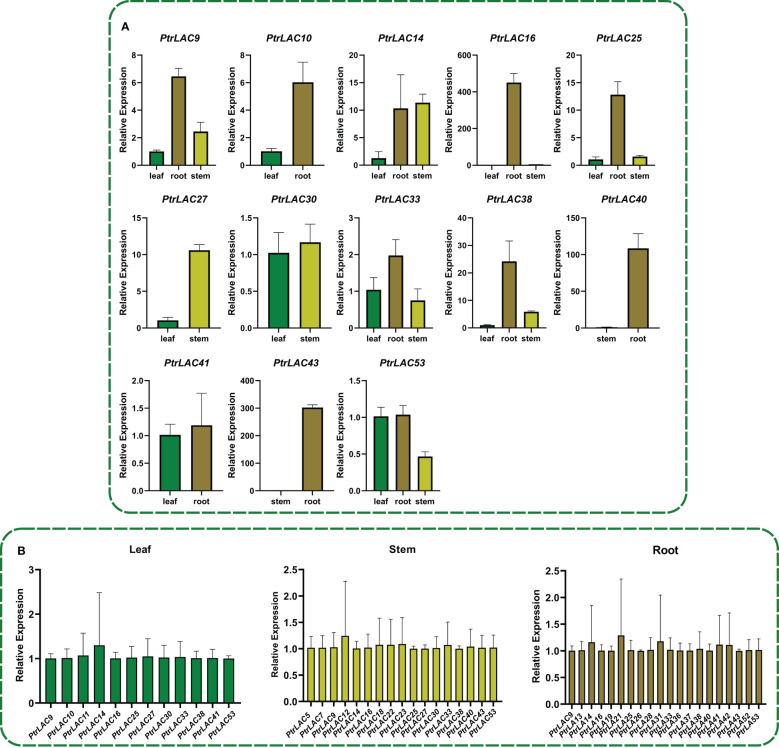
Expression analysis of *PtrLACs* by qRT-PCR. **(A)** Relative expression of *PtrLACs* in different tissues, Transcript levels in “leaf” were set to “1”, except *PtrLAC40*, *PtrLAC43*, which levels in stem were set to “1”. **(B)** Relative expression levels of *PtrLACs* in the same tissue. *PtrLAC9* was set to “1” in “Leaf” and “Root”, while *PtrLAC5* was set to “1” in “Stem”. All the data were normalized to *18S rRNA* genes, bars represent standard deviation.

In order to understand the relative expression level of *PtrLACs* in different tissues at the early stage of secondary growth, we selected 84K poplar (*P.alba×P.glandulosa*) trees which were 45-day-old, and analyzed the relative expression levels of *PtrLACs* in root, stem and leaf by Quantitative Real-time PCR. It turns out that only part of *PtrLACs* were detected in the early stage of secondary growth, including 20 genes in roots, 17 genes in stems and 12 genes in leaves. We also found some genes with spatial expression specificity in roots and stems, such as *PtrLAC5, PtrLAC7, PtrLAC23, PtrLAC12, PtrLAC18* and *PtrLAC22*, which are currently only expressed in stems. During this period, *PtrLAC13*, *PtrLAC19, PtrLAC21, PtrLAC26, PtrLAC28, PtrLAC31, PtrLAC36, PtrLAC37, PtrLAC42* and *PtrLAC52* were only expressed in roots. In addition, the expression levels of *PtrLAC* genes were relatively close in the same sample, no significant difference was detected. By comparing the relative expression of one *PtrLAC* in different samples, we found that the expression level of most *PtrLAC* genes was higher in roots than in leaves and stems. Expression levels of *PtrLAC9, PtrLAC10, PtrLAC16, PtrLAC25, PtrLAC33, PtrLAC40* and *PtrLAC43* in roots were significantly higher than those in other tissues. (**Figure 8**).

### 3.6 Cis-elements in PtrLACs promoters

**Figure 9:**
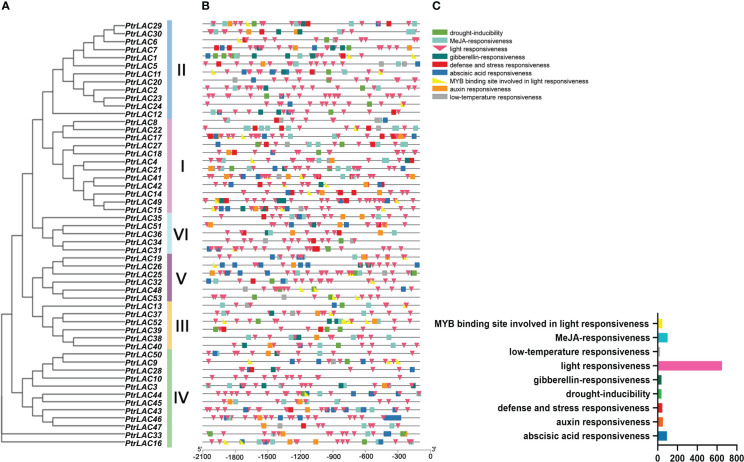
Predicted cis-elements in *PtrLAC* promoters. **(A)** Neighbor-joining tree of *PtrLAC* gene family **(B)** Promoter sequences (−2000 bp) of 53 *PtrLACs* were analyzed by PlantCARE. Diverse cis-acting elements were displayed in different colors and shapes. **(C)** Histogram of the number of different cis-acting elements. Cis-acting elements represented by different colors and shapes were shown in the top right-hand corner.

To further investigate the potential regulatory mechanisms of *PtrLACs*, we used PlantCARE to predict cis-acting elements within 2.0-kb upstream of each gene sequences, nine cis-acting elements were analyzed and displayed in **Figure 8**. We found that the number of cis-acting elements involved in photoresponse was significantly higher than others and some of them have *MYB* protein binding sites (Baldoni et al., 2015). In view of this interesting finding, we conjectured that the regulation of laccase expression is closely related to photosynthesis. Plastocyanin (Pc) is an important part of the electron transport chain of photosynthesis, which transfer electrons through the redox changes of copper ions in proteins (Höhner et al., 2020). The activity of laccase is also dependent on copper ion (Sun et al., 2019), which leads to the competition effect of copper ions between laccase and Pc. It has been reported that under copper ion stress, the transcriptional regulator SPL7 binds to the copper responsive cis-element (GTAC) in *miR397* promoter to promote *miR397* synthesis, thereby inhibiting the expression of laccase (Yamasaki et al., 2009). Therefore, we speculated that under copper ion stress, light may inhibit the expression of laccase genes, so that copper ion in plants can meet pc requirements and ensure the progress of photosynthesis. In addition, many *PtrLAC* photoresponsive cis-elements contain *MYB* protein binding sites, suggesting that *MYB* may be participate in regulation of this physiological process as a trans-factor. Besides, *PtrLACs* also possess cis-acting elements involved in plant hormone response, especially to jasmonic acid and abscisic acid, indicating that laccase may be related to inducing plant chemical defense and bud dormancy. We also detected the cis-elements related to drought induction, low temperature response, defense and stress response in *PtrLACs*, suggesting a potential stress response under certain conditions. Anyhow, the cis-element analysis illustrated that *PtrLACs* can respond to different abiotic factors, among which light and copper ion concentration may play a crucial role in the regulation of laccase expression. (**Figure 9**)

### 3.7 Structure modelling of representative PtrLACs

In order to explore the relationship between the structure and function of laccase proteins, we selected some representative laccases and simulated its 3D structure by using AlphaFold2 (Cramer, 2021). Older genes may be more closely related to the basic physiological functions in plants. Therefore, we selected *PtrLAC25*, a gene that had a collinear relationship with all five species in **Figure 6** and participated in fragment repetition 200 million years ago, and *PtrLAC41*, a gene that had a collinear relationship with multiple *PtrLACs* both in WGD events and 200 million years ago. *AtLAC2* has been suggested an involvement in dehydration response (Cai et al., 2006). *PtrLAC12* as a member of group 2 is likely to be involved in *Populus* developmental lignification. In phylogenetic analysis, *PtrLAC12* and *AtLAC2* had a strong genetic relationship and close genetic distance. Combined with the results of cis-acting elements prediction, we speculated that *PtrLAC12* may also played a role in drought stress. Therefore, as a potentially functionally complex laccase, *PtrLAC12* was used as a member of the structural simulation. The Cu-oxidase_3 domain of PtrLAC51 was severely lost, which was the most different from other laccase genes in sequence structure, so it was also within our selection range. *PtrLAC23* is also a member of group 2 and it was important to note that it did not participate in any repetition events of *PtrLAC* gene family and its sequence was complete, without motif loss. Meanwhile, it has been proved that *miRNA novel-m1190-3p* targets *PtrLAC23* in the secondary stem of *P. trichocarpa* (Wang et al., 2021), so we speculated that this gene may be related to wood formation, thus we also predict its structure.

ZmLAC3 is the only plant laccase with known structure and has been confirmed to be implicated in the polymerisation of lignin in maize. Structural comparison with ZmLAC3 protein show that five laccases (PtrLAC12, PtrLAC23, PtrLAC25, PtrLAC41, PtrLAC51) all have low RMSD values (< 3 Å) with ZmLAC3, which means their protein structures are almost the same with ZmLAC3 (Reva et al., 1998) (Pitera, 2014). *Populus* lignin is mainly composed of G and S type monomers, while H type monomer content is very low (Humphreys and Chapple, 2002). Therefore, coniferyl alcohol (ConA) and sinapyl alcohol (SinA) were selected for molecular docking with selected laccases. Results show that the selected five laccases generally prefer ConA to SinA, and PtrLAC23 has the highest affinity with ConA among them.

### 3.8 PtrLAC23 may be a lignin-related laccase in *Populus trichocarpa*

**Figure 10:**
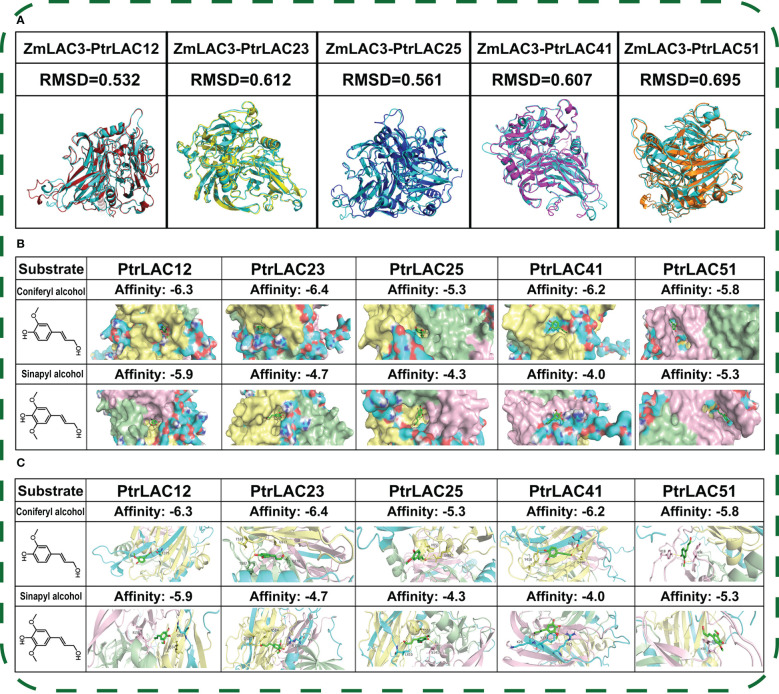
Structure modelling of PtrLAC12, PtrLAC23, PtrLAC25, PtrLAC41, PtrLAC51 by using AlphaFold2. **(A)** Structure comparison between PtrLAC and ZmLAC3, ZmLAC3 (cyan), PtrLAC12 (red), PtrLAC23 (yellow), PtrLAC25 (blue), PtrLAC41(magenta), PtrLAC51(orange); RMSD, Root Mean Squared Deviation. **(B)** Pocket shape of PtrLAC substrate binding site, proteins are shown in “surface” form. Pink region represent Cu-oxidase_3 domain, green region represent Cu-oxidase domain, yellow region represent Cu-oxidase_2 domain. Affinity, free-energy of enzyme-substrate binding (more negative value depicts better binding). **(C)** Polar interactions between substrate molecules and amino acids near the binding sites.

The results of **Figure 10** indicated that *PtrLAC23* (Potri.009G156600) may be involved in the lignification process of *Populus*. To further prove this prediction, we constructed the fusion protein expression vector of *PtrLAC23* and enhanced Green Fluorescent protein (EGFP) initiated by the endogenous promoter: *pLAC23-LAC23-EGFP*, which was introduced into 84K poplar by *Agrobacterium tumefaciens* to obtain stable genetic *proLAC23::LAC23-EGFP* transgenic material. Stem sections of the transgenic plants (growing for 2 months) were observed by LEICA DM2500 fluorescence microscope. Basic principles and flow of the above operations are shown in **Figure S1**.

**Figure 11:**
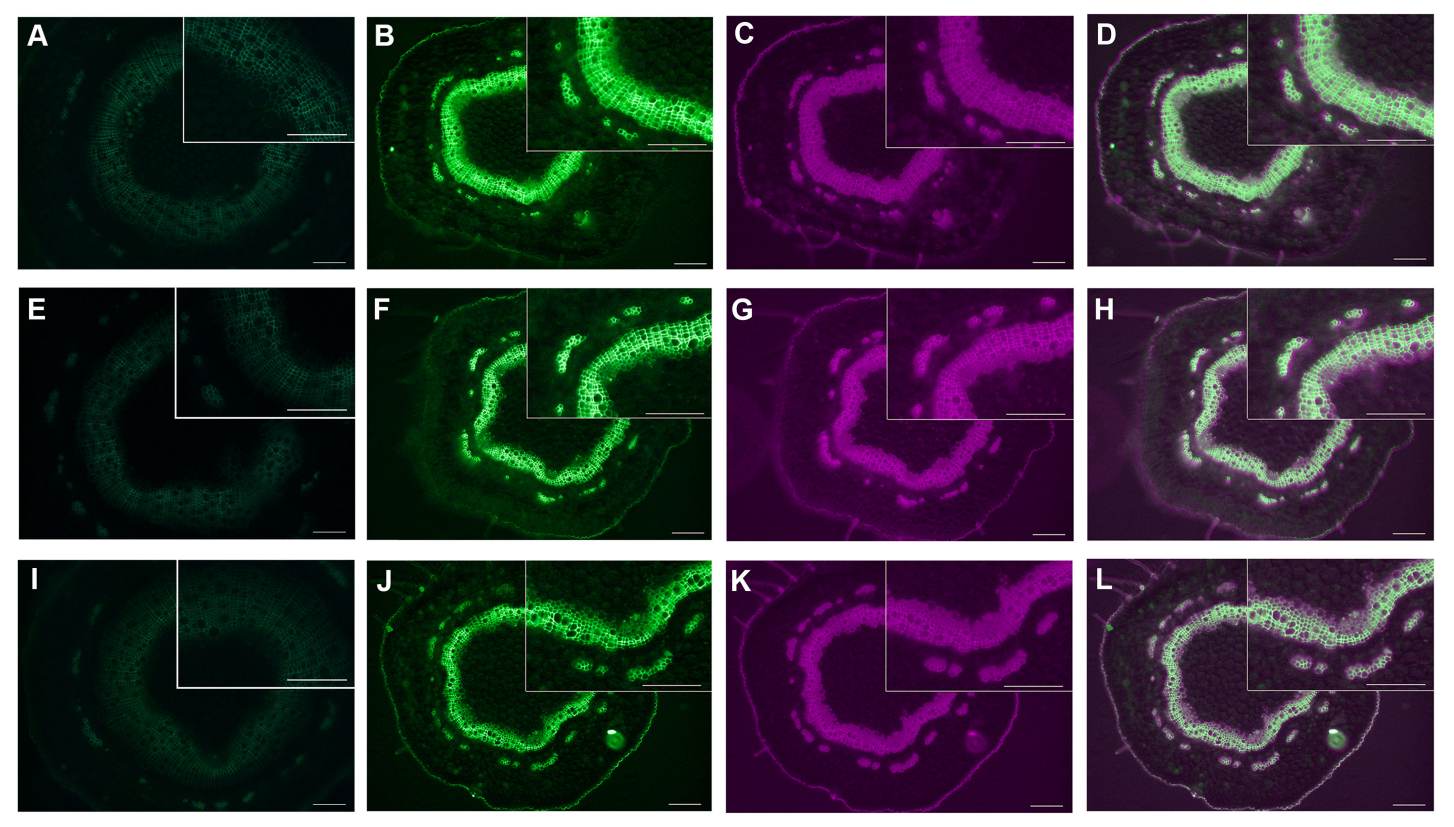
Expression and localization of *PtrLAC23* and lignin in different sections of 84k poplar stem. **(A, E, I)** Spontaneous fluorescence of wild-type poplar under blue excitation light.**(B, F, J)** Fluorescence of *pLAC23-LAC23-EGFP* fusion protein (green). **(C, G, K)** Fluorescence of lignin (magenta). **(D, H, L)** Fluorescence of *pLAC23-LAC23-EGFP* fusion protein and lignin. *Upper panel* shows the localization of *PtrLAC23* and lignin at 2.0 cm away from the bottom of the stem. Localization of *PtrLAC23* and lignin at 3.5 cm and 5.0 cm from the bottom of the stem are displayed in *middle and lower panels* respectively. Bar in **(A–L)** = 100 μm.

**Figure 12:**
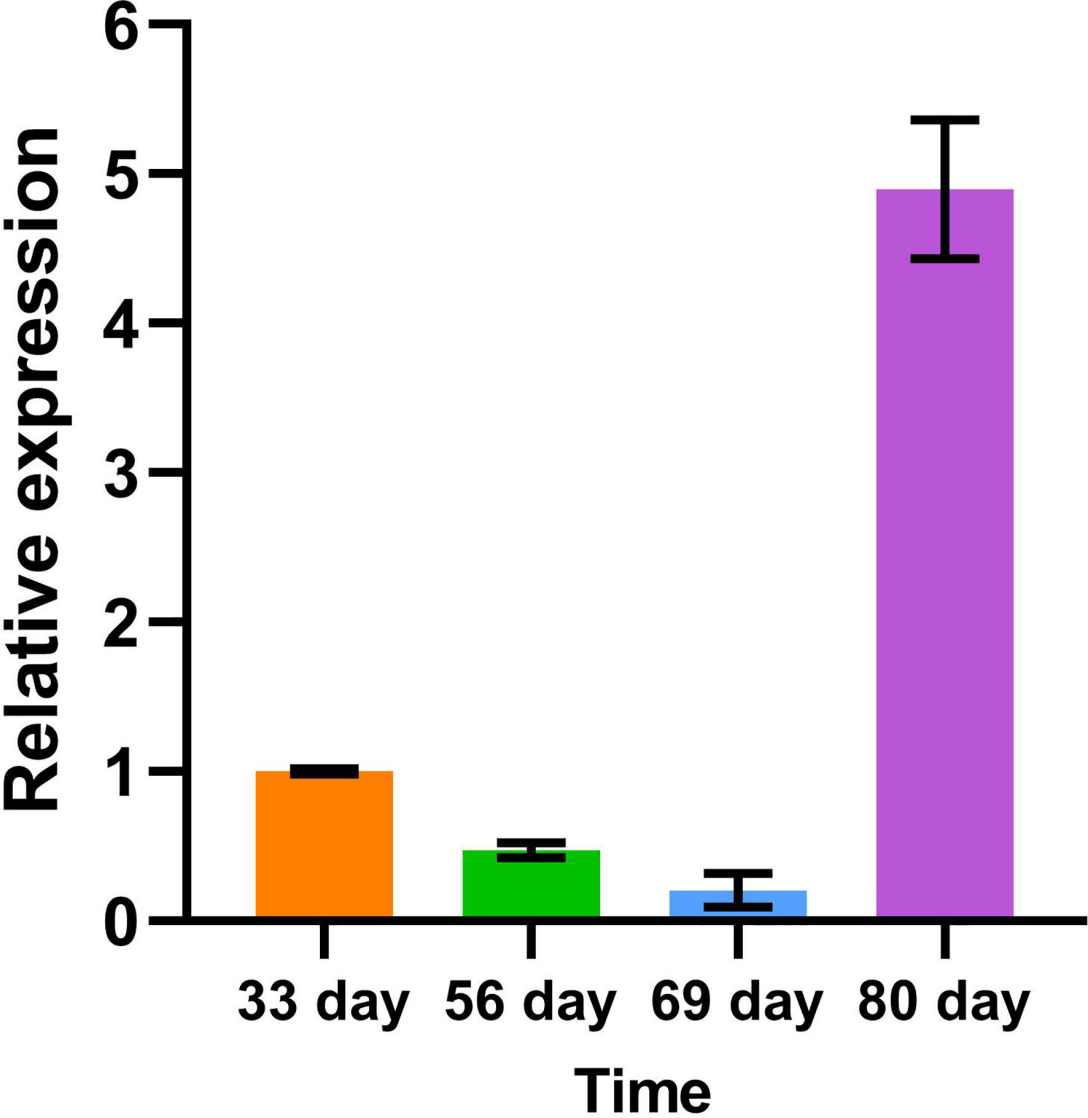
Relative expression of *PtrLAC23* in *Populus* stems at different growth stages.

We use the green fluorescence of EGFP and spontaneous fluorescence of lignin to locate *PtrLAC23* and lignin, respectively. It turns out that *PtrLAC23* is mainly located in xylem, and a small amount is distributed in phloem region. Its distribution position is almost the same as that of lignin, which can be inferred that *PtrLAC23* has a certain correlation with the synthesis of lignin. (**Figure 11**). To further verify the function of *PtrLAC23*, we determined the relative expression level of *PtrLAC23* in poplar stems at different growth stages. Results showed that compared with the 33 days, 56 days and 69 days samples, the expression level of *PtrLAC23* was significantly increased in stems of 80 day-old *Populus* (**Figure 12**).

## 4 Discussion

Laccases, as the largest subfamily of the polycopper oxidase (LMCOs) protein family, have a wide variety of substrates and are widely distributed in plants, insects and microorganisms. Previous studies have shown that plant laccase plays an important role in the oxidation and polymerization of lignin monomers. Under aerobic conditions, laccase can catalyze the oxidative dehydrogenation of lignin monomers to form free radicals, thus initiating the spontaneous polymerization process between monomers. Therefore, the regulation of plant laccase gene expression can indirectly regulate the composition and content of lignocellulose, which provides an effective basis for the improvement of energy trees. *Populus trichocarpa* is a model plant in forest research. Although its laccase gene family has been identified, detailed analysis of family members was still relatively scarce.

Gene duplication is one of the main causes of gene family formation. There were 22 genes involved in tandem duplication and 22 genes involved in fragment duplication in *Populus* laccase gene family, indicating that the two gene duplication events played an equally important role in the expansion of *PtrLAC* gene family. In addition, the Ka/Ks values of almost all *PtrLAC* duplicate gene pairs were less than 1, illustrating that *PtrLACs* were underwent a strong purifying selective pressure during the evolution. This conclusion is consistent with the results obtained in *Pyrus bretschneideri* (Cheng X et al., 2020), *Glycine max* (Wang et al., 2019) and *Setaria viridis* (Simões MS et al., 2020), suggesting that this may be a general rule in the plant kingdom. Combined with the molecular evolution rate of *Populus*, we estimated the replication time of collinear gene pairs and found that fragment duplication and tandem duplication were the main driving force of gene family expansion in the early and late stages, respectively. In addition, most *PtrLACs* were replicate after the WGD event, this is different from the results in soybean, which laccase genes replication typically occured during WGD events (Wang et al., 2019). This result also shows that the laccase gene family has different expansion modes in different species. Moreover, 20% of the duplicate gene pairs were separated before 150 MYA, indicating that laccase genes have a very long history in *Populus*, which also allows us to Preliminary lock a series of ancient *PtrLACs* represented by *PtrLAC25* and *PtrLAC41*.

In the early study, *Arabidopsis* laccase gene family was divided into 6 subfamilies, and most of the subsequent studies were based on this criterion. In our study, PtrLACs were distributed in all six subfamilies, in which gene duplication was the main cause of the expansion in group 4, group 5, and group 6. The function of acidic or basic isoforms of laccase in lignification has always been controversial, it is generally believed that acidic peroxidases have poor ability to catalyze sinapyl alcohol (Barceló AR et al., 2007). The evolutionary analysis also revealed that acidic PtrLACs mainly clustered within group 3 and group 4, which happened to be the group of ZmLAC1 and ZmLAC3, respectively. Moreover, ZmLAC3 as an acidic laccase has been confirmed to be involved in injury-induced lignification in maize. Therefore, we hypothesized that the acidic laccases in *Populus* may be functionally specific, and the acidic laccases belonging to the group 3 may also play a role in the process of defense-induced lignification. Although, ZmLAC2, ZmLAC4, and ZmLAC5, which belong to the group 2 have not been confirmed to be involved in the process of injury-induced lignification, their expression patterns were similar to the one expected for genes involved in lignification (Caparrós-Ruiz et al., 2006). Therefore, we speculated that they might play a role in the developmental lignification, and PtrLACs in group 2 may also potentially involved in this process. In conclusion, we believe that PtrLACs in group 3 and 4 may be involved in the defense-induced lignification in *Populus*, and PtrLACs in Group 2 may be involved in the developmental lignification in *Populus*.

Motif is generally considered to be conserved sequence with biological functions, which may contain specific binding sites or common sequence segments involved in a specific biological process. Adjacent *PtrLACs* on the evolutionary tree generally have more similar gene structure and motif composition. However, a few of members in group 4 and group 6 have lost part of motifs, indicating that these laccases may have acquired new functions in the evolution. In fact, loss events of genes occur frequently during the evolution. Due to the whole-genome triplication (WGT), genes in *Arabidopsis* should have three homologs in *B. rapa* and *B. oleracea*, but in fact, some *AtLACs* can not even have homologs in either species (Lu et al., 2019). The genome of *Populus* is about four times as large as that of *Arabidopsis*, while the number of laccase genes in *Populus* is only three times as large as in *Arabidopsis*. All of these phenomena indicate that gene loss events existed in *LAC* gene family universally.

A large number of studies have shown that laccase exists widely in plants and is of great significance for maintaining plant growth and development. Synteny analysis between *Populus* and five other species showed that some *PtrLACs* (*PtrLAC11, PtrLAC25, PtrLAC20, PtrLAC19*) may have existed before the divergence of monocots and dicots ancestors. Combined with the estimation of the replication time of fragment duplication genes, we found that *PtrLAC25* is not only a relatively old gene in *Populus*, but also has homology with laccase genes in five other species, which deserves more attention.

Gene expression pattern can reflect the function of genes to a certain extent. RNA-Seq data showed that most *PtrLAC* genes were highly expressed in nodes, internodes and catkins, which again indicated the important role of laccase in the polymerization of lignin monomers. Lu et al. have found that 49 laccase genes were all expressed in the 6-month-old *Populus*. However, we could only detect the expression of a few of laccase genes in the early stage of *Populus* secondary growth. *PtrLACs* (*PtrLAC5, PtrLAC7, PtrLAC23, PtrLAC12, PtrLAC18* and *PtrLAC22*) specifically expressed in roots and stems are both members of group 1 and group 2, which is consistent with the findings in *Setaria* (Simões MS et al., 2020). Among them, *PtrLAC5*, *PtrLAC7*, *PtrLAC23* and *PtrLAC12* are both members of group 2. The specific expression of these genes in stem, a lignin-accumulating tissue, again proves the conclusion that members of group 2 may be related to the developmental lignification in *Populus*. In addition, expression level of some *PtrLAC* genes were significantly higher in roots. These laccase genes, with the exception of *PtrLAC25*, all belong to group 3 and group 4. Cotton laccase GhLAC1 is also preferentially expressed in roots. Previous studies have shown that it was involved in the lignification process induced by defense and thus improves the resistance of cotton to biotic stress (Acid et al., 2018).Therefore, we believe that these *PtrLACs*, which are mainly expressed in roots at the initial stage of secondary growth, may also play a potential role in plant defense besides participating in root lignification. This also suggests that members of group 3 and group 4 are likely to be involved in defense-induced lignification in *Populus*.

It has been shown that there is a dynamic equilibrium between photosynthesis and cell wall synthesis, and copper ion concentration in plants is the key to regulate this equilibrium. When copper ions are deficient in plants, *miRNA* (*AtmiR397*, *AtmiR398*, *AtmiR408*, *AtmiR857* in *Arabidopsis*; *PtmiR397*, *PtmiR408*, *PtmiR1444* in *Populus*) will inhibit the expression of other copper-containing proteins, and preferentially supply copper to the essential copper-containing proteins in the photosynthetic electron transport chain represented by PC. Cis-acting elements prediction showed that *PtrLAC* genes had a large number of photoresponsiveness cis-acting elements. In addition, the activity of laccase also depends on the copper ion center, which suggests that there is a relationship between photosynthesis, copper ion concentration and the regulation of laccase gene expression. We also found that MYB protein may be involved in this process as a transcription factor. The network mechanism of MYB family members regulating plant secondary cell wall formation has been fully revealed. However, there are still few reports on the interaction between *MYB* protein and laccase, which needs to be verified by subsequent studies (Xiao et al., 2021).

Understanding the structure of a protein is crucial for defining its function. With the development of artificial intelligence (AI), AlphaFold2 made a breakthrough in the protein folding problem (Marcu et al., 2022). In CASP14, AlphaFold2 achieved a remarkable result. Some of its predicted protein structures were almost indistinguishable from the experimental results (Flower and Hurley, 2021). ZmLAC3 in maize is the only plant laccase which structure has been resolved. Moreover, ZmLAC3 has been proved to catalyze the oxidative polymerization of lignin monomers. The structure prediction of five *Populus* laccase proteins showed that the overall structure of the PtrLAC12, PtrLAC23, PtrLAC25, PtrLAC41 and PtrLAC51 is very close to that of ZmLAC3. These findings indicate that laccase is structurally conserved, and suggest that these proteins may play a potential role in the polymerization of lignin monomers. In addition, PtrLAC23 has the strongest affinity to lignin monomer ConA, which may be a key laccase related to lignification in *Populus*.

At present, there is a lot of evidence that laccase is involved in cell wall lignification in *Populus*. Bryan et al. found that *PdLAC2* plays a role in overcoming plant cell wall recalcitrance in *Populus deltoides* (Bryan et al., 2016). Liu et al. showed that PtrLAC16 can polymerize lignin monomers *in vitro*, inhibition the expression of *PtrLAC16* led to a significant decrease in lignin content and altered cell wall structure (Liu et al., 2021). Niu et al. proved that overexpression of *PeuLAC2* increased the secondary cell wall thickening, fiber cell length and stem tensile strength (Niu et al., 2021). Tissue specificity analysis of these laccases showed that *PtrLAC16* is specifically localized in the xylem and phloem of the stem. Expression of *PeuLAC2* in *P. euphratica* tissues mainly occurred in the xylem. In *P. deltoides*, *LAC2* had the highest expression in xylem compared to other tissues. In our study, fluorescence observation of cross sections of *proLAC23::LAC23-EGFP* line showed that the expression localization of *PtrLAC23* was mainly in xylem and a few areas of the phloem, and was highly consistent with the deposition site of lignin. These results are similar to the tissue localization analysis of laccases above, which again proved the correlation between *PtrLAC23* and *Populus* wood formation. In addition, relative expression of *PtrLAC23* was significantly increased in the stem of 80-day-old *Populus* than that of young *Populus*, suggesting that the role of *PtrLAC23* in *Populus* stem’s secondary growth should not be ignored. The predicted results showed that *PtrLAC23* was likely an extracellular protein. Subcellular localization of PtrLAC16 and PeuLAC2 both showed that laccase protein was probably secreted extracellular and transported to the cell wall to play a role (Niu et al., 2021) (Liu et al., 2021). Here, we also observed the fluorescence of proLAC23::LAC23-EGFP transgenic *Populus* protoplasts (**Figure S2**). In our results, only chloroplast spontaneous fluorescence was observed and no EGFP signal was found, which also confirmed that PtrLAC23 should be an extracellular protein that probably be transported to play a role on the cell wall.

## 5 Conclusions

In summary, sequence characteristics, structures and functions of the 53 members of the *PtrLAC* gene family have been elucidated in detail. They can be divided into 6 subfamilies according to their phylogenetic relationships with *Arabidopsis* and maize. Gene duplication analysis indicated that some colinear gene pairs separated 200 million years ago. Moreover, in the process of gene family expansion, fragment repetition is the main driving force in the early stage while tandem repetition plays this role in the late stage. Most laccase proteins are likely to be extracellular, but some have been shown to localize to cell membranes or to lysosomes and peroxisomes. Gene structure and motif analysis demonstrated that adjacent members in the phylogenetic tree had similar intro-exon structure and motif composition. Domain analysis, expression pattern analysis and cis-acting elements prediction shed light on the complex function of *Populus* laccase. Protein structure simulation and molecular docking analysis of PtrLAC12, PtrLAC23, PtrLAC25, PtrLAC41 and PtrLAC51 indicated that PtrLAC23likely to involved in the lignification process. Fluorescence observation of *proLAC23:: LAC23-EGFP* transgenic *Populus* stem transects showed that the location of PtrLAC23 highly overlapped with the lignin deposition. This indicates that PtrLAC23 may play an important role in *Populus* lignin synthesis. Plant laccase is of great significance to improve the utilization efficiency of lignocellulose. Regulating the expression of plant laccase can indirectly influence the content of lignin and cellulose, which can provide the theoretical support for us to obtain ideal biomass materials. These results also provides a broad prospect for us to obtain more ideal biofuels through genetic engineering in the future.

## Data availability statement

The datasets presented in this study can be found in online repositories. The names of the repository/repositories and accession number(s) can be found in the article/**Supplementary Material**.

## Author contributions

BL conceived the idea, performed the experimental work and wrote the original draft; CW and XL contributed to data curation, review and editing original draft, YM contributed to the design, supervision and direction of experiment, HR provided equipment and technical support for the operation of AlphaFold2, YZ Contributed to project management, material provision and financial support. All authors contributed to the article and approved the submitted version.
