# Supplementary material for: Genome-wide analysis of the Populus trichocarpa laccase gene family and functional identification of PtrLAC23
Source: Front Plant Sci. 2023 Jan 17;13:1063813. doi: 10.3389/fpls.2022.1063813 (PMC9887407; doi:10.3389/fpls.2022.1063813)
Supplement: Supplementary file 1 [file DataSheet_1.pdf]

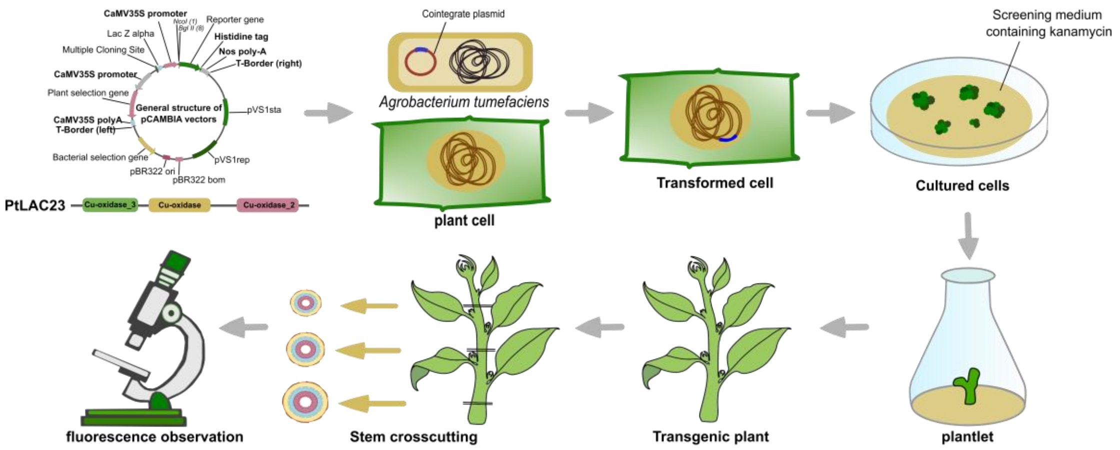

Fig. S1 Schematic diagram of experimental process of agrobacterium-mediated transformation (ATMT), transgenic plant screening, freehand section and fluorescence observation.
