# Supplementary material for: Genome-wide analysis of the Populus trichocarpa laccase gene family and functional identification of PtrLAC23
Source: Front Plant Sci. 2023 Jan 17;13:1063813. doi: 10.3389/fpls.2022.1063813 (PMC9887407; doi:10.3389/fpls.2022.1063813)
Supplement: Supplementary file 2 [file DataSheet_2.pdf]

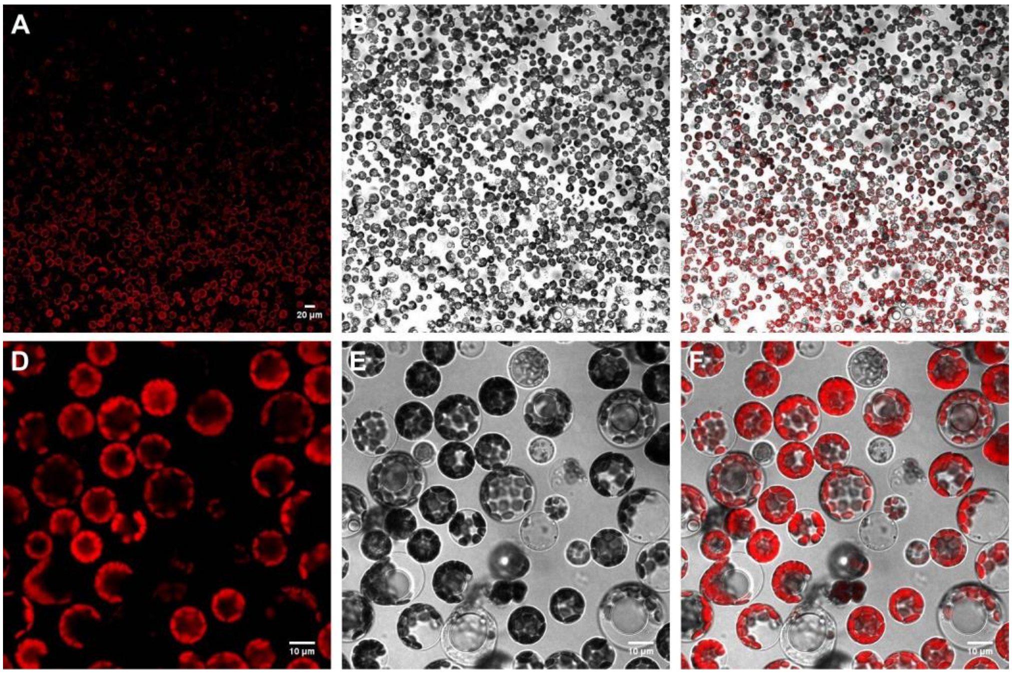

Fig. S2. Fluorescence observation of *proLAC23::LAC23-EGFP* transgenic *Populus* protoplasts. (A) spontaneous fluorescence of chloroplast at 20×; (B) image under the bright field at 20×; (C) image after the merge of (A) and (B); (D) spontaneous fluorescence of chloroplast at 100×; (E) image under the bright field at 100×; (F) image after the merge of (D) and (E).
