# Supplementary material for: Genome-wide analysis of the Populus trichocarpa laccase gene family and functional identification of PtrLAC23
Source: Front Plant Sci. 2023 Jan 17;13:1063813. doi: 10.3389/fpls.2022.1063813 (PMC9887407; doi:10.3389/fpls.2022.1063813)
Supplement: Supplementary file 3 [file Table_1.docx]

**Table S1. Primers used for qRT-PCR analysis**

| Gene name | Forward primer | Reverse primer |
| --- | --- | --- |
| *PtrLAC1* | ATTAATGGACTCCCAGGGCCATTA | TTCACATACACAGCATCAGCTTCC |
| *PtrLAC2* | AGTTTTGAGTTTCACCAATATTACCTG | CCAGAGGCAGCTCATAGCAAAT |
| *PtrLAC3* | TCACCATCAATGGAGAACCAGGATA | GCCAGGGGTGATCATTAGATAGTCT |
| *PtrLAC4* | AGGGAGGATATAATTTACCAGTACGG | CAATGGTGTCGATTTTGAAGGGTTTA |
| *PtrLAC5* | GGCAAGACTTACATGCTCCGCTTA | AAACAGCATCAACATCAACAATGGTA |
| *PtrLAC6* | CTCTGATGCCTACACTATCAATGGAT | TGGTTTCACATAAATAGCATCAACAC |
| *PtrLAC7* | CTCTGATGCCTACACTATCAATGGAT | GTTTGCTATGCTGAAGAAGAGCTCG |
| *PtrLAC8* | CATACCATTAATGGGAAGCCAGGGA | CAATCAGTACTGCTTGAGTGGTAAAAT |
| *PtrLAC9* | CATTCACTGGCATGGAGTGAAACAA | GCCACCATAATGTTCCTTCTTCGTT |
| *PtrLAC10* | GAGACACAATCTATGTTACGGTCCAT | CCAGGTTGGATTGGACACTGTGTA |
| *PtrLAC11* | GGCCTTCCAGGGCCTTTATACAAT | CTGGACTGATGAGGAGCGTGTCA |
| *PtrLAC12* | CCAGGAAAAACCTATCTCCTTCGTT | CGGGGTGATGAGTACAATGTGGGT |
| *PtrLAC13* | CTACCCTTGCTCTCAAAACAGAATATT | CGATGACAACAACGCCAGTGACG |
| *PtrLAC14* | TGCCTCTTCCCTGCTTTGGTCC | GCCATTGACGGTAACAATCGGC |
| *PtrLAC15* | TCTTGCTGTCTGCCTCCTCCCC | GCCATTGACGGTAACAATCGGC |
| *PtrLAC16* | TACTAGTGATGCCATCTACAGG | TTGCCCTTTCCATAGCCTA |
| *PtrLAC17* | CATACTTTTGCAATGGAGATTGAATCA | ATCTGCCTGGAACTTGGTTGGCTA |
| *PtrLAC18* | ACCTCCCAGTGACCTCCCCAAC | CTTTGTTTGAGCACTTGAGCCCAG |
| *PtrLAC19* | GGTCAACCTGGTGATCTTTTTAACTGC | AACTGTAAACTTGTGGTTGGCTATG |
| *PtrLAC20* | GCTGATCCTGAGGCAGTGATTAGA | AAGGTTGGTCTCAAAAGGCTTCACA |
| *PtrLAC21* | CACGGAGGGTATAATTTATCAGTACAT | CAACGGTGTCGATTTTGAAGGGTTTT |
| *PtrLAC22* | ATACATTTACCTTGGAGGTTGAACAG | CAATTAGTATGGTTTGAGTTGTAAAGG |
| *PtrLAC23* | CGAGGCACTACAAGTTCAACATAG | CCATTAACAGTCACCATGCTCTTC |
| *PtrLAC24* | AGTTTTGAGTTTCACCAATAGTACCTA | CCAGAGGCAGCTCATAGCAAAG |
| *PtrLAC25* | CTTGGTCGAGAATGGAGTCGGAG | GCTTATGAATTTCAACAAACTGGTACG |
| *PtrLAC26* | GGTCAACCTGGTGATCTTTATAATTGT | TAAGATAAGAGGCATCAGCACCAATA |
| *PtrLAC27* | ATGAGTCAATACTACCACCTCCT | CTATACTCCTCCCTCCCGCATC |
| *PtrLAC28* | CACAAGACTCTGCAGCACCAAGAA | GGCTGTTTCACTCCATGCCAGTGA |
| *PtrLAC29* | AGGCCCAAATGTCTCTGATGCTTAT | AAGAAGAGCTCGTCATTGAGTGCG |
| *PtrLAC30* | GCAGATCCAGAGGCGATCATTAGT | AAGAAGAGCTCGTCATTGAGGGCA |
| *PtrLAC31* | ATGCCGTATCCATTCTCAGCTCAG | GGCATTCCATTTATGGTGTAAGCGT |
| *PtrLAC32* | GTAATCTTCAGTTTCCAAGCTCATTT | TTTGATCACAAGAGTATCGCCATCG |
| *PtrLAC33* | GGCACCTTCTTCTGGCATGCTCAT | AGATGTACAATATCCTGAAGCCAGTA |
| *PtrLAC34* | GGCGTTACCGTGTTGTTCTTCAC | CAATCCGATTTTTGACCTTAATATGAATAC |
| *PtrLAC35* | ACGAAGCAGCTTCTAATGGTGAAC | TCTGAGCAATCCGATTTTTGACC |
| *PtrLAC36* | GGGATCCAGATGAAGTTGAAAACAG | GCTATCGCAAAGAAGAGCTCATCC |
| *PtrLAC37* | TGTTGATGTCGAAAACCAGGCAGAA | GATTGGCTATCTTGAAAAAGAGCTGG |
| *PtrLAC38* | GCTTCTGCTGCAATTGTGGAACG | AAGCGTTGGGCCTGGCAAGCTG |
| *PtrLAC39* | GATCCTGTCAACGACCATAAGAAT | CCAGATGACAGTGAAAGAACCAC |
| *PtrLAC40* | GCTCATTCTATGTGAAAAACCTTACAG | AGCGTTGGGCCTGGCAAGCTT |
| *PtrLAC41* | AAGGCGGTTTCACTTTGCCAGTCC | TAGGTGGCATCAACTTCCACGACA |
| *PtrLAC42* | GGGCGGTTTCACATTGCCAGTCG | GTAGGTGGCATCAACTTCCACAAG |
| *PtrLAC43* | ACAATTATTATTGGATCTTGGTTTAAGG | CATTGATGGTAAGACTGTTGGATATATT |
| *PtrLAC44* | GAAATCAACACTGTTGCACTTCG | GGCGCATACTTTGTGCCGTTGTAT |
| *PtrLAC45* | ACAATTATTATTGGATCTTGGTTTAAGG | CATTGATGGTAAGACTGTTGGATATATT |
| *PtrLAC46* | ACAATTATTATTGGATCTTGGTTTAAGC | CCATTGATGGTAAGACTATTGGATGG |
| *PtrLAC47* | GCAACTGGTGCTGGACCTGCAAT | TTAACCTTCAAACGGTATGTGTTTTCT |
| *PtrLAC48* | GTAATCATCAGTTTCCAAGCATCTTC | CTTGATCACAAGAGTATCACCGTCA |
| *PtrLAC49* | CAGCAAATATGGAGTACTCCAATTG | TGCCGAATCCTGCACTCGACC |
| *PtrLAC50* | ATGTCTCTGATTCTTACACGA | GTAAGTCTTGCCATAGTCCAC |
| *PtrLAC51* | ATACTTTTATTCAGACGGTGG | CTGTCAACGTATGTTTCGCTA |
| *PtrLAC52* | ACATTTCGGATGCCTACACCA | CCCTTTCTGCACCTTAAGCTTG |
| *PtrLAC53* | TATAACTCAGCCAATGCACCT | TCCCTTTGTTGGCATTGCAAG |
| *18s rRNA* | TCAACTTTCGATGGTAGGATAGAG | CCGTGTCAGGATTGGGTAATTT |

**Table S2 Ka/Ks analysis and doubling time estimation of fragment repeat genes in *PtrLAC* gene family**

| Seq_1 | Seq_2 | Ka | Ks | Ka/Ks | Doubling time(MYA) |
| --- | --- | --- | --- | --- | --- |
| *PtrLAC4* | *PtrLAC14* | 0.128930303 | 2.159725 | 0.059698 | 53.6723468 |
| *Potri.001G243200.1* | *PtrLAC20* | 0.078863467 | 0.268362 | 0.29387 | 49.8866088 |
| *PtrLAC4* | *PtrLAC21* | 0.036832278 | 0.249433 | 0.147664 | 63.368719 |
| *PtrLAC6* | *PtrLAC29* | 0.051781883 | 0.316844 | 0.16343 | 380.3721894 |
| *PtrLAC4* | *PtrLAC41* | 0.129968213 | 1.901861 | 0.068337 | 53.6597946 |
| *Potri.005G079400.1* | *PtrLAC16* | 0.061266496 | 0.268299 | 0.228352 | 61.0611178 |
| *PtrLAC13* | *PtrLAC37* | 0.059322702 | 0.305306 | 0.194306 | 62.802885 |
| *PtrLAC14* | *PtrLAC41* | 0.029230409 | 0.314014 | 0.093086 | 1.0919066 |
| *Potri.007G088300.1* | *PtrLAC16* | 0 | 0.00546 | 0 | 58.9985688 |
| *PtrLAC18* | *PtrLAC27* | 0.047500248 | 0.294993 | 0.161022 | 208.1545118 |
| *PtrLAC19* | *PtrLAC25* | 0.100059247 | 1.040773 | 0.096139 | 384.0464416 |
| *PtrLAC18* | *PtrLAC41* | 0.208936236 | 1.920232 | 0.108808 | 380.3518462 |
| *PtrLAC27* | *PtrLAC41* | 0.197092755 | 1.901759 | 0.103637 | 61.3872806 |
| *PtrLAC31* | *PtrLAC34* | 0.061552134 | 0.306936 | 0.200537 | 47.891737 |
| *PtrLAC32* | *PtrLAC53* | 0.047858078 | 0.239459 | 0.199859 | 2.423316 |
| *PtrLAC48* | *PtrLAC53* | 0.000766 | 0.012117 | 0.063226 |  |

**Table S3 Ka/Ks analysis and doubling time estimation of tandem repeat genes in *PtrLAC* gene family**

| Seq_1 | Seq_2 | Ka | Ks | Ka/Ks | Doubling time(MYA) |
| --- | --- | --- | --- | --- | --- |
| *PtrLAC50* | *PtrLAC9* | 0.016415 | 0.04924 | 0.333373 | 9.848096 |
| *PtrLAC9* | *PtrLAC10* | 0.264266 | 1.4214 | 0.185919 | 284.2801 |
| *PtrLAC14* | *PtrLAC15* | 0.012018 | 0.027332 | 0.439697 | 5.466443 |
| *PtrLAC15* | *PtrLAC49* | 0.003977 | 0.017256 | 0.230448 | 3.451262 |
| *PtrLAC29* | *PtrLAC30* | 0.006856 | 0.046861 | 0.146301 | 9.372269 |
| *PtrLAC25* | *PtrLAC26* | 0.105338 | 0.881274 | 0.11953 | 176.2549 |
| *PtrLAC35* | *PtrLAC36* | 0.030201 | 0.047409 | 0.63702 | 9.481829 |
| *PtrLAC36* | *PtrLAC51* | 0.01908 | 0.032894 | 0.580051 | 6.578722 |
| *PtrLAC37* | *PtrLAC38* | 0.028398 | 0.047451 | 0.598458 | 9.490292 |
| *PtrLAC41* | *PtrLAC42* | 0.023066 | 0.161139 | 0.143146 | 32.22772 |
| *PtrLAC43* | *PtrLAC44* | 0.021525 | 0.050537 | 0.425925 | 10.10745 |
| *PtrLAC44* | *PtrLAC45* | 0.012854 | 0.031893 | 0.403044 | 6.378512 |
| *PtrLAC45* | *PtrLAC46* | 0.028069 | 0.021106 | 1.329937 | 4.221172 |
| *PtrLAC46* | *PtrLAC47* | 0.014031 | 0.020267 | 0.692297 | 4.053445 |

**Table S4 General properties of *P. trichocarpa* laccase gene family members**

| Name | Gene ID | AA | MW (kDa) | pI | Signal Peptide Length | Cleavage Site | Subcellular localization |
| --- | --- | --- | --- | --- | --- | --- | --- |
| *PtrLAC1* | Potri.001G054600.1 | 581 | 64.262 | 9.29 | 31 | AVA-TT | Extracellular, Lysosomal |
| *PtrLAC2* | Potri.001G184300.1 | 576 | 63.529 | 9.33 | 31 | VAG-AK | Extracellular, Lysosomal |
| *PtrLAC3* | Potri.001G206200.1 | 559 | 62.859 | 9.21 | 24 | ANG-KI | Lysosomal, Peroxisomal |
| *PtrLAC4* | Potri.001G248700.1 | 557 | 61.145 | 9.05 | 24 | VES-MV | Extracellular |
| *PtrLAC5* | Potri.001G341600.1 | 580 | 64.163 | 8.91 | 30 | AFA-VT | Extracellular, Lysosomal |
| *PtrLAC6* | Potri.001G401100.1 | 581 | 64.264 | 9.19 | 31 | AIA-TT | Lysosomal |
| *PtrLAC7* | Potri.001G401300.1 | 581 | 64.197 | 9.2 | 31 | ALA-TT | Extracellular, Lysosomal |
| *PtrLAC8* | Potri.004G156400.1 | 564 | 62.557 | 8.94 | 30 | AKA-AV | Extracellular, Lysosomal |
| *PtrLAC9* | Potri.005G200600.1 | 566 | 63.558 | 7.62 | 26 | CQA-IV | Extracellular, Lysosomal |
| *PtrLAC10* | Potri.005G200700.1 | 565 | 63.026 | 6.5 | 25 | CLA-AT | Extracellular, Plasma Membrane, Lysosomal |
| *PtrLAC11* | Potri.006G087100.1 | 580 | 63.79 | 9.19 | 33 | AAA-TT | Extracellular, Lysosomal |
| *PtrLAC12* | Potri.006G087500.1 | 579 | 64.094 | 9.84 | 25 | VVA-KH | Peroxisomal |
| *PtrLAC13* | Potri.006G094100.1 | 562 | 61.508 | 8.3 | 22 | ASA-AI | Extracellular, Plasma Membrane, Lysosomal |
| *PtrLAC14* | Potri.006G096900.1 | 558 | 61.311 | 9.52 | 23 | VQC-RV | Extracellular, Lysosomal |
| *PtrLAC15* | Potri.006G097000.1 | 559 | 61.285 | 9.56 | 24 | VEC-RI | Extracellular, Plasma Membrane, Lysosomal |
| *PtrLAC16* | Potri.T079500.1 | 582 | 65.597 | 7.32 | 35 | AEA-RI | Extracellular, Plasma Membrane |
| *PtrLAC17* | Potri.007G023300.1 | 562 | 62.356 | 7.26 | 28 | AEA-AI | Lysosomal |
| *PtrLAC18* | Potri.008G064000.1 | 556 | 60.948 | 7.27 | 23 | VEC-EV | Plasma Membrane |
| *PtrLAC19* | Potri.008G073700.1 | 574 | 63.889 | 9.23 | 30 | AIA-KT | Extracellular, Plasma Membrane |
| *PtrLAC20* | Potri.009G034500.1 | 576 | 63.523 | 9.35 | 31 | VAG-AK | Extracellular, Lysosomal |
| *PtrLAC21* | Potri.009G042500.1 | 556 | 60.74 | 9.03 | 23 | VES-MV | Extracellular |
| *PtrLAC22* | Potri.009G102700.1 | 561 | 62.753 | 8.91 | 27 | AEA-AV | Lysosomal, Peroxisomal |
| *PtrLAC23* | Potri.009G156600.1 | 576 | 63.512 | 9.2 | 31 | VAG-AK | Extracellular, Lysosomal |
| *PtrLAC24* | Potri.009G156800.1 | 576 | 63.481 | 9.31 | 31 | VAG-AK | Extracellular, Lysosomal |
| *PtrLAC25* | Potri.010G183500.1 | 582 | 64.164 | 7.62 | 34 | ANA-KS | Extracellular |
| *PtrLAC26* | Potri.010G183600.1 | 575 | 63.672 | 8.75 | 31 | AIA-EI | Extracellular, Plasma Membrane |
| *PtrLAC27* | Potri.010G193100.1 | 555 | 60.919 | 8.72 | 22 | VEC-KV | Extracellular, Plasma Membrane |
| *PtrLAC28* | Potri.011G071100.1 | 562 | 63.079 | 6.67 | 22 | CQA-IV | Extracellular |
| *PtrLAC29* | Potri.011G120200.1 | 581 | 63.935 | 9.03 | 31 | ALA-IT | Extracellular, Lysosomal |
| *PtrLAC30* | Potri.011G120300.1 | 581 | 64.027 | 9.18 | 31 | ALA-IT | Extracellular, Lysosomal |
| *PtrLAC31* | Potri.012G048900.1 | 579 | 64.456 | 9.35 | 27 | CSS-QA | Plasma Membrane, Lysosomal |
| *PtrLAC32* | Potri.013G152700.1 | 576 | 64.491 | 9.26 | 32 | SEA-ET | Extracellular, Plasma Membrane, Lysosomal |
| *PtrLAC33* | Potri.014G100600.1 | 572 | 63.456 | 7.26 | 24 | IHA-AP | Extracellular |
| *PtrLAC34* | Potri.015G040400.1 | 579 | 64.38 | 8.52 | 27 | CSS-QT | Extracellular, Lysosomal |
| *PtrLAC35* | Potri.015G040600.1 | 579 | 64.499 | 8.96 | 27 | CSS-ET | Lysosomal |
| *PtrLAC36* | Potri.015G040700.1 | 540 | 60.058 | 9.23 | 27 | CSS-QT | Plasma Membrane, Lysosomal |
| *PtrLAC37* | Potri.016G106000.1 | 568 | 61.924 | 6.82 | 22 | ASA-AI | Extracellular, Plasma Membrane, Lysosomal |
| *PtrLAC38* | Potri.016G106100.1 | 569 | 62.029 | 8.05 | 22 | ASA-AI | Plasma Membrane, Lysosomal |
| *PtrLAC39* | Potri.016G106300.1 | 562 | 61.924 | 6.82 | 22 | ASA-AI | Extracellular, Plasma Membrane, Lysosomal |
| *PtrLAC40* | Potri.016G107900.1 | 569 | 61.583 | 8.31 | 22 | ASA-AI | Plasma Membrane, Lysosomal |
| *PtrLAC41* | Potri.016G112000.1 | 557 | 60.916 | 9.3 | 22 | VEC-RI | Extracellular, Plasma Membrane |
| *PtrLAC42* | Potri.016G112100.1 | 564 | 62.027 | 9.53 | 29 | VEC-RV | Extracellular, Plasma Membrane |
| *PtrLAC43* | Potri.019G088500.1 | 567 | 62.76 | 7.01 | 26 | CMA-QS | Plasma Membrane |
| *PtrLAC44* | Potri.019G088600.1 | 567 | 62.781 | 7.29 | 26 | CMA-QS | Plasma Membrane |
| *PtrLAC45* | Potri.019G088700.1 | 567 | 62.77 | 7.68 | 26 | CMA-QS | Plasma Membrane |
| *PtrLAC46* | Potri.019G088800.1 | 567 | 62.919 | 6.78 | 26 | CMA-QS | Plasma Membrane |
| *PtrLAC47* | Potri.019G088900.1 | 567 | 62.919 | 6.78 | 26 | CMA-QS | Plasma Membrane |
| *PtrLAC48* | Potri.019G121700.1 | 576 | 63.9 | 8.23 | 29 | ASS-SE | Extracellular, Plasma Membrane, Lysosomal |
| *PtrLAC49* | Potri.006G097100.1 | 559 | 61.462 | 9.56 | 24 | VEC-RI | Extracellular, Plasma Membrane, Lysosomal |
| *PtrLAC50* | Potri.005G200500.1 | 566 | 63.588 | 7.04 | 26 | CQA-IV | Extracellular, Plasma Membrane, Lysosomal |
| *PtrLAC51* | Potri.015G040800.1 | 480 | 53.343 | 8.91 |  |  | Plasma Membrane, Lysosomal |
| *PtrLAC52* | Potri.016G107500.1 | 568 | 61.958 | 6.82 | 22 | ASA-AI | Extracellular, Plasma Membrane, Lysosomal |
| *PtrLAC53* | Potri.019G124300.1 | 576 | 63.888 | 8.23 | 29 | ASS-SE | Extracellular, Plasma Membrane, Lysosomal |
