# Supplementary material for: Genome-wide analysis of the Populus trichocarpa laccase gene family and functional identification of PtrLAC23
Source: Front Plant Sci. 2023 Jan 17;13:1063813. doi: 10.3389/fpls.2022.1063813 (PMC9887407; doi:10.3389/fpls.2022.1063813)
Supplement: Supplementary file 8 [file Table_6.docx]

Colinear pairs of laccase genes between *P. trichocarpa* and *S. viridis*:

spe694-Chr_05 spe694-Sevir.5G371400.1.v2.1 == spe555-Chr08 spe555-Potri.008G073700.1.v3.0

spe694-Chr_05 spe694-Sevir.5G371400.1.v2.1 == spe555-Chr10 spe555-Potri.010G183500.1.v3.0

spe694-Chr_05 spe694-Sevir.5G388000.1.v2.1 == spe555-Chr16 spe555-Potri.016G106100.1.v3.0

spe694-Chr_05 spe694-Sevir.5G371400.1.v2.1 == spe555-Chr19 spe555-Potri.019G124300.1.v3.0

spe694-Chr_08 spe694-Sevir.8G016500.1.v2.1 == spe555-Chr10 spe555-Potri.010G183500.1.v3.0

spe694-Chr_08 spe694-Sevir.8G016500.1.v2.1 == spe555-Chr19 spe555-Potri.019G124300.1.v3.0

spe694-Chr_09 spe694-Sevir.9G457500.1.v2.1 == spe555-Chr01 spe555-Potri.001G243200.1.v3.0

spe694-Chr_09 spe694-Sevir.9G457500.1.v2.1 == spe555-Chr06 spe555-Potri.006G087100.1.v3.0

spe694-Chr_09 spe694-Sevir.9G457500.1.v2.1 == spe555-Chr09 spe555-Potri.009G034500.1.v3.0

spe694-Chr_09 spe694-Sevir.9G440800.1.v2.1 == spe555-Chr10 spe555-Potri.010G183500.1.v3.0

Colinear pairs of laccase genes between *P. trichocarpa* and *A. thaliana*:

Chr1 AT1G18140.1.TAIR10 == Chr12 Potri.012G048900.1.v3.0

Chr1 AT1G18140.1.TAIR10 == Chr15 Potri.015G040400.1.v3.0

Chr2 AT2G38080.1.TAIR10 == Chr06 Potri.006G096900.1.v3.0

Chr2 AT2G29130.1.TAIR10 == Chr06 Potri.006G087100.1.v3.0

Chr2 AT2G40370.1.TAIR10 == Chr08 Potri.008G073700.1.v3.0

Chr2 AT2G30210.1.TAIR10 == Chr08 Potri.008G073700.1.v3.0

Chr2 AT2G29130.1.TAIR10 == Chr09 Potri.009G034500.1.v3.0

Chr2 AT2G40370.1.TAIR10 == Chr10 Potri.010G183500.1.v3.0

Chr2 AT2G30210.1.TAIR10 == Chr10 Potri.010G183600.1.v3.0

Chr2 AT2G30210.1.TAIR10 == Chr13 Potri.013G152700.1.v3.0

Chr2 AT2G46570.1.TAIR10 == Chr14 Potri.014G100600.1.v3.0

Chr2 AT2G38080.1.TAIR10 == Chr16 Potri.016G112000.1.v3.0

Chr2 AT2G30210.1.TAIR10 == Chr19 Potri.019G121700.1.v3.0

Chr3 AT3G09220.1.TAIR10 == Chr06 Potri.006G094100.1.v3.0

Chr3 AT3G09220.1.TAIR10 == Chr16 Potri.016G107500.1.v3.0

Chr5 AT5G58910.1.TAIR10 == Chr01 Potri.001G248700.1.v3.0

Chr5 AT5G01190.1.TAIR10 == Chr06 Potri.006G096900.1.v3.0

Chr5 AT5G58910.1.TAIR10 == Chr06 Potri.006G097000.1.v3.0

Chr5 AT5G05390.1.TAIR10 == Chr08 Potri.008G073700.1.v3.0

Chr5 AT5G58910.1.TAIR10 == Chr09 Potri.009G042500.1.v3.0

Chr5 AT5G05390.1.TAIR10 == Chr10 Potri.010G183500.1.v3.0

Chr5 AT5G01190.1.TAIR10 == Chr16 Potri.016G112000.1.v3.0

Colinear pairs of laccase genes between *P. trichocarpa* and *G.max*:

spe698-Chr01 spe698-Glyma.01G112600.1.Wm82.a2.v1 == spe893-Chr01 spe893-Potri.001G248700.1.v3.0

spe698-Chr01 spe698-Glyma.01G173500.1.Wm82.a2.v1 == spe893-Chr04 spe893-Potri.004G156400.1.v3.0

spe698-Chr01 spe698-Glyma.01G108200.1.Wm82.a2.v1 == spe893-Chr06 spe893-Potri.006G094100.1.v3.0

spe698-Chr01 spe698-Glyma.01G112600.1.Wm82.a2.v1 == spe893-Chr06 spe893-Potri.006G096900.1.v3.0

spe698-Chr01 spe698-Glyma.01G173500.1.Wm82.a2.v1 == spe893-Chr07 spe893-Potri.007G023300.1.v3.0

spe698-Chr01 spe698-Glyma.01G183100.1.Wm82.a2.v1 == spe893-Chr07 spe893-Potri.007G038200.1.v3.0

spe698-Chr01 spe698-Glyma.01G112600.1.Wm82.a2.v1 == spe893-Chr09 spe893-Potri.009G042500.1.v3.0

spe698-Chr01 spe698-Glyma.01G108200.1.Wm82.a2.v1 == spe893-Chr16 spe893-Potri.016G107500.1.v3.0

spe698-Chr01 spe698-Glyma.01G112600.1.Wm82.a2.v1 == spe893-Chr16 spe893-Potri.016G112000.1.v3.0

spe698-Chr01 spe698-Glyma.01G108200.1.Wm82.a2.v1 == spe893-Chr16 spe893-Potri.016G106000.1.v3.0

spe698-Chr02 spe698-Glyma.02G224800.1.Wm82.a2.v1 == spe893-Chr01 spe893-Potri.001G248700.1.v3.0

spe698-Chr02 spe698-Glyma.02G261600.1.Wm82.a2.v1 == spe893-Chr08 spe893-Potri.008G073700.1.v3.0

spe698-Chr02 spe698-Glyma.02G224800.1.Wm82.a2.v1 == spe893-Chr09 spe893-Potri.009G042500.1.v3.0

spe698-Chr02 spe698-Glyma.02G261600.1.Wm82.a2.v1 == spe893-Chr10 spe893-Potri.010G183500.1.v3.0

spe698-Chr02 spe698-Glyma.02G231600.1.Wm82.a2.v1 == spe893-Chr13 spe893-Potri.013G152700.1.v3.0

spe698-Chr02 spe698-Glyma.02G231600.1.Wm82.a2.v1 == spe893-Chr19 spe893-Potri.019G121700.1.v3.0

spe698-Chr02 spe698-Glyma.02G231600.1.Wm82.a2.v1 == spe893-Chr19 spe893-Potri.019G124300.1.v3.0

spe698-Chr03 spe698-Glyma.03G077900.1.Wm82.a2.v1 == spe893-Chr06 spe893-Potri.006G096900.1.v3.0

spe698-Chr03 spe698-Glyma.03G077900.1.Wm82.a2.v1 == spe893-Chr16 spe893-Potri.016G112000.1.v3.0

spe698-Chr04 spe698-Glyma.04G019500.1.Wm82.a2.v1 == spe893-Chr04 spe893-Potri.004G180500.1.v3.0

spe698-Chr04 spe698-Glyma.04G019500.1.Wm82.a2.v1 == spe893-Chr05 spe893-Potri.005G247700.1.v3.0

spe698-Chr07 spe698-Glyma.07G225300.1.Wm82.a2.v1 == spe893-Chr01 spe893-Potri.001G000500.1.v3.0

spe698-Chr07 spe698-Glyma.07G261800.1.Wm82.a2.v1 == spe893-Chr04 spe893-Potri.004G010100.1.v3.0

spe698-Chr07 spe698-Glyma.07G133900.1.Wm82.a2.v1 == spe893-Chr06 spe893-Potri.006G087100.1.v3.0

spe698-Chr07 spe698-Glyma.07G134100.1.Wm82.a2.v1 == spe893-Chr06 spe893-Potri.006G087500.1.v3.0

spe698-Chr07 spe698-Glyma.07G142400.1.Wm82.a2.v1 == spe893-Chr06 spe893-Potri.006G094100.1.v3.0

spe698-Chr07 spe698-Glyma.07G054100.1.Wm82.a2.v1 == spe893-Chr14 spe893-Potri.014G100600.1.v3.0

spe698-Chr07 spe698-Glyma.07G142400.1.Wm82.a2.v1 == spe893-Chr16 spe893-Potri.016G106000.1.v3.0

spe698-Chr08 spe698-Glyma.08G359100.1.Wm82.a2.v1 == spe893-Chr01 spe893-Potri.001G054600.1.v3.0

spe698-Chr08 spe698-Glyma.08G138900.1.Wm82.a2.v1 == spe893-Chr07 spe893-Potri.007G088300.1.v3.0

spe698-Chr08 spe698-Glyma.08G343500.1.Wm82.a2.v1 == spe893-Chr14 spe893-Potri.014G177700.1.v3.0

spe698-Chr10 spe698-Glyma.10G219100.1.Wm82.a2.v1 == spe893-Chr05 spe893-Potri.005G200500.1.v3.0

spe698-Chr10 spe698-Glyma.10G197300.1.Wm82.a2.v1 == spe893-Chr08 spe893-Potri.008G032100.1.v3.0

spe698-Chr10 spe698-Glyma.10G197300.1.Wm82.a2.v1 == spe893-Chr10 spe893-Potri.010G229500.1.v3.0

spe698-Chr10 spe698-Glyma.10G219100.1.Wm82.a2.v1 == spe893-Chr19 spe893-Potri.019G088500.1.v3.0

spe698-Chr11 spe698-Glyma.11G069500.1.Wm82.a2.v1 == spe893-Chr07 spe893-Potri.007G023300.1.v3.0

spe698-Chr11 spe698-Glyma.11G233400.1.Wm82.a2.v1 == spe893-Chr08 spe893-Potri.008G073700.1.v3.0

spe698-Chr11 spe698-Glyma.11G137500.1.Wm82.a2.v1 == spe893-Chr09 spe893-Potri.009G034500.1.v3.0

spe698-Chr11 spe698-Glyma.11G233400.1.Wm82.a2.v1 == spe893-Chr10 spe893-Potri.010G183500.1.v3.0

spe698-Chr11 spe698-Glyma.11G164000.1.Wm82.a2.v1 == spe893-Chr13 spe893-Potri.013G152700.1.v3.0

spe698-Chr11 spe698-Glyma.11G164000.1.Wm82.a2.v1 == spe893-Chr19 spe893-Potri.019G121700.1.v3.0

spe698-Chr12 spe698-Glyma.12G060900.1.Wm82.a2.v1 == spe893-Chr09 spe893-Potri.009G034500.1.v3.0

spe698-Chr12 spe698-Glyma.12G192800.1.Wm82.a2.v1 == spe893-Chr14 spe893-Potri.014G154500.1.v3.0

spe698-Chr14 spe698-Glyma.14G191500.1.Wm82.a2.v1 == spe893-Chr01 spe893-Potri.001G248700.1.v3.0

spe698-Chr14 spe698-Glyma.14G041300.1.Wm82.a2.v1 == spe893-Chr01 spe893-Potri.001G219300.1.v3.0

spe698-Chr14 spe698-Glyma.14G056100.1.Wm82.a2.v1 == spe893-Chr08 spe893-Potri.008G073700.1.v3.0

spe698-Chr14 spe698-Glyma.14G062300.1.Wm82.a2.v1 == spe893-Chr08 spe893-Potri.008G064000.1.v3.0

spe698-Chr14 spe698-Glyma.14G191500.1.Wm82.a2.v1 == spe893-Chr09 spe893-Potri.009G042500.1.v3.0

spe698-Chr14 spe698-Glyma.14G041300.1.Wm82.a2.v1 == spe893-Chr09 spe893-Potri.009G159700.1.v3.0

spe698-Chr14 spe698-Glyma.14G056100.1.Wm82.a2.v1 == spe893-Chr10 spe893-Potri.010G183500.1.v3.0

spe698-Chr14 spe698-Glyma.14G062300.1.Wm82.a2.v1 == spe893-Chr10 spe893-Potri.010G193100.1.v3.0

spe698-Chr14 spe698-Glyma.14G198900.1.Wm82.a2.v1 == spe893-Chr13 spe893-Potri.013G152700.1.v3.0

spe698-Chr14 spe698-Glyma.14G198900.1.Wm82.a2.v1 == spe893-Chr19 spe893-Potri.019G121700.1.v3.0

spe698-Chr14 spe698-Glyma.14G198900.1.Wm82.a2.v1 == spe893-Chr19 spe893-Potri.019G124300.1.v3.0

spe698-Chr16 spe698-Glyma.16G158400.1.Wm82.a2.v1 == spe893-Chr05 spe893-Potri.005G200500.1.v3.0

spe698-Chr17 spe698-Glyma.17G261500.1.Wm82.a2.v1 == spe893-Chr05 spe893-Potri.005G247700.1.v3.0

spe698-Chr18 spe698-Glyma.18G065100.1.Wm82.a2.v1 == spe893-Chr01 spe893-Potri.001G248700.1.v3.0

spe698-Chr18 spe698-Glyma.18G177300.1.Wm82.a2.v1 == spe893-Chr01 spe893-Potri.001G054600.1.v3.0

spe698-Chr18 spe698-Glyma.18G183500.1.Wm82.a2.v1 == spe893-Chr06 spe893-Potri.006G087100.1.v3.0

spe698-Chr18 spe698-Glyma.18G183700.1.Wm82.a2.v1 == spe893-Chr06 spe893-Potri.006G087500.1.v3.0

spe698-Chr18 spe698-Glyma.18G193200.1.Wm82.a2.v1 == spe893-Chr06 spe893-Potri.006G094100.1.v3.0

spe698-Chr18 spe698-Glyma.18G023600.1.Wm82.a2.v1 == spe893-Chr08 spe893-Potri.008G073700.1.v3.0

spe698-Chr18 spe698-Glyma.18G065100.1.Wm82.a2.v1 == spe893-Chr09 spe893-Potri.009G042500.1.v3.0

spe698-Chr18 spe698-Glyma.18G023600.1.Wm82.a2.v1 == spe893-Chr10 spe893-Potri.010G183500.1.v3.0

spe698-Chr18 spe698-Glyma.18G057200.1.Wm82.a2.v1 == spe893-Chr13 spe893-Potri.013G152700.1.v3.0

spe698-Chr18 spe698-Glyma.18G193200.1.Wm82.a2.v1 == spe893-Chr16 spe893-Potri.016G106000.1.v3.0

spe698-Chr18 spe698-Glyma.18G193400.1.Wm82.a2.v1 == spe893-Chr16 spe893-Potri.016G106300.1.v3.0

spe698-Chr18 spe698-Glyma.18G057200.1.Wm82.a2.v1 == spe893-Chr19 spe893-Potri.019G121700.1.v3.0

spe698-Chr20 spe698-Glyma.20G025200.1.Wm82.a2.v1 == spe893-Chr01 spe893-Potri.001G000500.1.v3.0

spe698-Chr20 spe698-Glyma.20G172600.1.Wm82.a2.v1 == spe893-Chr05 spe893-Potri.005G200500.1.v3.0

spe698-Chr20 spe698-Glyma.20G192800.1.Wm82.a2.v1 == spe893-Chr08 spe893-Potri.008G032100.1.v3.0

spe698-Chr20 spe698-Glyma.20G126500.1.Wm82.a2.v1 == spe893-Chr10 spe893-Potri.010G114200.1.v3.0

spe698-Chr20 spe698-Glyma.20G192800.1.Wm82.a2.v1 == spe893-Chr10 spe893-Potri.010G229500.1.v3.0

spe698-Chr20 spe698-Glyma.20G172600.1.Wm82.a2.v1 == spe893-Chr19 spe893-Potri.019G088500.1.v3.0

spe698-scaffold_27 spe698-Glyma.U027300.1.Wm82.a2.v1 == spe893-Chr06 spe893-Potri.006G094100.1.v3.0

spe698-scaffold_27 spe698-Glyma.U027300.1.Wm82.a2.v1 == spe893-Chr16 spe893-Potri.016G106000.1.v3.0

Colinear pairs of laccase genes between *P. trichocarpa* and *O.sativa*:

chr03 Os03t0273200-01 == Chr06 Potri.006G087100.1.v3.0

chr03 Os03t0297900-00 == Chr10 Potri.010G183500.1.v3.0

chr05 Os05t0458300-00 == Chr06 Potri.006G087100.1.v3.0

chr11 Os11t0108650-00 == Chr10 Potri.010G183500.1.v3.0

chr11 Os11t0108650-00 == Chr13 Potri.013G152700.1.v3.0

chr11 Os11t0108650-00 == Chr19 Potri.019G124300.1.v3.0

chr01 Os01t0843800-00 == Chr06 Potri.006G087500.1.v3.0

chr01 Os01t0850550-00 == Chr06 Potri.006G094100.1.v3.0

chr01 Os01t0827300-01 == Chr13 Potri.013G152700.1.v3.0

chr01 Os01t0850550-00 == Chr16 Potri.016G107500.1.v3.0

chr01 Os01t0827300-01 == Chr19 Potri.019G124300.1.v3.0

chr12 Os12t0108000-01 == Chr10 Potri.010G183500.1.v3.0

chr12 Os12t0108000-01 == Chr13 Potri.013G152700.1.v3.0

Colinear pairs of laccase genes between *P. trichocarpa* and *S.bicolor*:

spe136-Chr01 spe136-Sobic.001G403100.1.v3.2 == spe561-Chr08 spe561-Potri.008G073700.1.v3.0

spe136-Chr03 spe136-Sobic.003G357500.1.v3.2 == spe561-Chr06 spe561-Potri.006G094100.1.v3.0

spe136-Chr03 spe136-Sobic.003G353200.1.v3.2 == spe561-Chr09 spe561-Potri.009G034500.1.v3.0

spe136-Chr03 spe136-Sobic.003G341500.1.v3.2 == spe561-Chr13 spe561-Potri.013G152700.1.v3.0

spe136-Chr03 spe136-Sobic.003G357500.1.v3.2 == spe561-Chr16 spe561-Potri.016G107500.1.v3.0

spe136-Chr08 spe136-Sobic.008G006900.1.v3.2 == spe561-Chr08 spe561-Potri.008G073700.1.v3.0

spe136-Chr08 spe136-Sobic.008G006900.1.v3.2 == spe561-Chr10 spe561-Potri.010G183500.1.v3.0
